# Consensus embedding: theory, algorithms and application to segmentation and classification of biomedical data

**DOI:** 10.1186/1471-2105-13-26

**Published:** 2012-02-08

**Authors:** Satish Viswanath, Anant Madabhushi

**Affiliations:** 1 Dept. of Biomedical Engineering, Rutgers University, 599 Taylor Road, Piscataway, New Jersey 08854, USA

## Abstract

**Background:**

Dimensionality reduction (DR) enables the construction of a lower dimensional space (embedding) from a higher dimensional feature space while preserving object-class discriminability. However several popular DR approaches suffer from sensitivity to choice of parameters and/or presence of noise in the data. In this paper, we present a novel DR technique known as consensus embedding that aims to overcome these problems by generating and combining multiple low-dimensional embeddings, hence exploiting the variance among them in a manner similar to ensemble classifier schemes such as Bagging. We demonstrate theoretical properties of consensus embedding which show that it will result in a single stable embedding solution that preserves information more accurately as compared to any individual embedding (generated via DR schemes such as Principal Component Analysis, Graph Embedding, or Locally Linear Embedding). Intelligent sub-sampling (via mean-shift) and code parallelization are utilized to provide for an efficient implementation of the scheme.

**Results:**

Applications of consensus embedding are shown in the context of classification and clustering as applied to: (1) image partitioning of white matter and gray matter on 10 different synthetic brain MRI images corrupted with 18 different combinations of noise and bias field inhomogeneity, (2) classification of 4 high-dimensional gene-expression datasets, (3) cancer detection (at a pixel-level) on 16 image slices obtained from 2 different high-resolution prostate MRI datasets. In over 200 different experiments concerning classification and segmentation of biomedical data, consensus embedding was found to consistently outperform both linear and non-linear DR methods within all applications considered.

**Conclusions:**

We have presented a novel framework termed consensus embedding which leverages ensemble classification theory within dimensionality reduction, allowing for application to a wide range of high-dimensional biomedical data classification and segmentation problems. Our generalizable framework allows for improved representation and classification in the context of both imaging and non-imaging data. The algorithm offers a promising solution to problems that currently plague DR methods, and may allow for extension to other areas of biomedical data analysis.

## Background

The analysis and classification of high-dimensional biomedical data has been significantly facilitated via the use of dimensionality reduction techniques, which allow classifier schemes to overcome issues such as the *curse of dimensionality*. This is an issue where the number of variables (features) is disproportionately large compared to the number of training instances (objects) [1]. Dimensionality reduction (DR) involves the projection of data originally represented in a *N*-dimensional (*N*-D) space into a lower *n*-dimensional (*n*-D) space (known as an *embedding*) such that *n << N*. DR techniques are broadly categorized as linear or non-linear, based on the type of projection method used.

Linear DR techniques make use of simple linear projections and consequently linear cost functions. An example of a linear DR scheme is Principal Component Analysis [2] (PCA) which projects data objects onto the axes of maximum variance. However, maximizing the variance within the data best preserves class discrimination only when distinct separable clusters are present within the data, as shown in [3]. In contrast, non-linear DR involves a non-linear mapping of the data into a reduced dimensional space. Typically these methods attempt to project data so that relative local adjacencies between high dimensional data objects, rather than some global measure such as variance, are best preserved during data reduction from *N*- to *n*-D space [4]. This tends to better retain class-discriminatory information and may also account for any non-linear structures that exist in the data (such as manifolds), as illustrated in [5]. Examples of these techniques include locally linear embedding [5] (LLE), graph embedding [6] (GE), and isometric mapping [7] (ISOMAP). Recent work has shown that in several scenarios, classification accuracy may be improved via the use of non-linear DR schemes (rather than linear DR) for gene-expression data [4,8] as well as medical imagery [9,10].

However, typical DR techniques such as PCA, GE, or LLE may not guarantee an optimum result due to one or both of the following reasons:

• Noise in the original *N*-D space tends to adversely affect class discrimination, even if robust features are used (as shown in [11]). A single DR projection may also fail to account for such artifacts (demonstrated in [12,13]).

• Sensitivity to choice of parameters being specified during projection; e.g. in [14] it was shown that varying the neighborhood parameter in ISOMAP can lead to significantly different embeddings.

In this paper, we present a novel DR scheme known as *consensus embedding *which aims to overcome the problems of sensitivity to noise and choice of parameters that plague several popular DR schemes [12-14]. The spirit behind consensus embedding is to construct a single stable embedding by generating and combining multiple uncorrelated, independent embeddings; the hypothesis being that this single stable embedding will better preserve specific types of information in the data (such as class-based separation) as compared to any of the individual embeddings. Consensus embedding may be used in conjunction with either linear or non-linear DR methods and, as we will show, is intended to be easily generalizable to a large number of applications and problem domains. In this work, we will demonstrate the superiority of the consensus embedding representation for a variety of classification and clustering applications.

**Figure 1:**
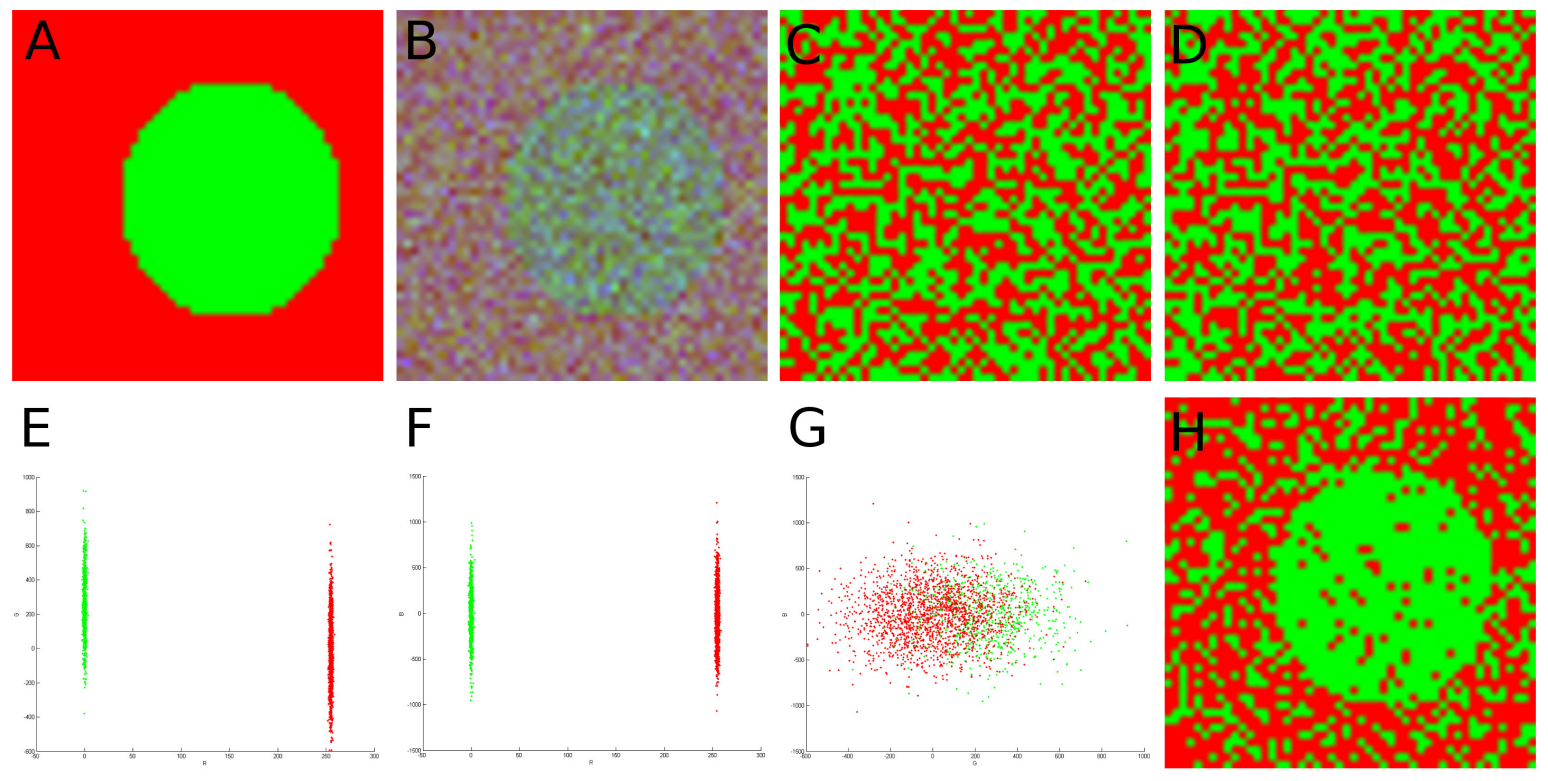
**Region partitioning of toy image data**. (a) Original RGB image to which Gaussian noise was added to create (b) noisy RGB image. Image visualization of classes obtained by replicated *k*-means clustering [15] of all the pixels via (c) original noisy RGB space, and (d) graph embedding [6] of noisy RGB data. 2D plots of (e) **R**-**G**, (f) **R**-**B**, and (g)**G**-**B **planes are also shown where colors of objects plotted correspond to the region in (b) that they are derived from. The discriminatory 2D spaces ((e) and (f)) are combined via consensus embedding, and the visualized classification result is shown in (h). Note the significantly better image partitioning into foreground and background of (h) compared to (c) and (d).

Figure 1 illustrates an application of consensus embedding in separating foreground (green) and background (red) regions via pixel-level classification. Figure 1(a) shows a simple RGB image to which Gaussian noise was added to the **G **and **B **color channels (see Figure 1(b)). We now consider each of the 3 color channels as features (i.e. *N *= 3) for all of the image objects (pixels). Classification via replicated *k*-means clustering [15] of all the objects (without considering class information) was first performed using the noisy RGB feature information (Figure 1(b)), in order to distinguish the foreground from background.

The labels so obtained for each object (pixel) are then visualized in the image shown in Figure 1(c), where the color of the pixel corresponds to its cluster label. The 2 colors in Figure 1(c) hence correspond to the 2 classes (clusters) obtained. No discernible regions are observable in this figure. Application of DR (via GE) reduces the data to a *n *= 2-D space, where the graph embedding algorithm [6] non-linearly projects the data such that the object classes are maximally discriminable in the reduced dimensional space. However, as seen in Figure 1(d), clustering this reduced embedding space does not yield any obviously discernible image partitions either.

By plotting all the objects onto 2D plots using only the **R**-**G **(Figure 1(e)) and **R**-**B **(Figure 1(f)) color channels respectively, we can see that separation between the two classes exists only along the **R **axis. In contrast, the 2D **G**-**B **plot (Figure 1(g)) shows no apparent separation between the classes. Combining 1D embeddings obtained via applying graph embedding to Figures 1(e) and 1(f), followed by unsupervised clustering, yields the consensus embedding result shown in Figure 1(h). Consensus embedding clearly results in superior background/foreground partitioning compared to the results shown in Figures 1(c),(d).

### Related Work and Significance

#### Classifier and clustering ensembles

Researchers have attempted to address problems of classifier sensitivity to noise and choice of parameters via the development of classifier ensemble schemes, such as Boosting [16] and Bagging [17]. These classifier ensembles guarantee a lower error rate as compared to any of the individual members (known as "weak" classifiers), assuming that the individual weak classifiers are all uncorrelated [18]. Similarly a consensus-based algorithm has been presented [15] to find a stable unsupervised clustering of data using unstable methods such as *k*-means [19]. Multiple "uncorrelated" clusterings of the data were generated and used to construct a co-association matrix based on cluster membership of all the points in each clustering. Naturally occurring partitions in the data were then identified. This idea was further extended in [20] where a combination of clusterings based on simple linear transformations of high-dimensional data was considered. Note that ensemble techniques thus (1) make use of uncorrelated, or relatively independent, analyses (such as classifications or projections) of the data, and (2) combine multiple analyses (such as classifications or projections) to enable a more stable result.

#### Improved DR schemes to overcome parameter sensitivity

As shown by [7], linear DR methods such as classical multi-dimensional scaling [21] are unable to account for non-linear proximities and structures when calculating an embedding that best preserves pairwise distances between data objects. This led to the development of non-linear DR methods such as LLE [5] and ISOMAP [7] which make use of local neighborhoods to better calculate such proximities. As previously mentioned, DR methods are known to suffer from certain shortcomings (sensitivity to noise and/or change in parameters). A number of techniques have recently been proposed to overcome these shortcomings.

In [22,23] methods were proposed to choose the optimal neighborhood parameter for ISOMAP and LLE respectively. This was done by first constructing multiple embeddings based on an intelligently selected subset of parameter values, and then choosing the embedding with the minimum residual variance. Attempts have been made to overcome problems due to noisy data by selecting data objects known to be most representative of their local neighborhood (landmarks) in ISOMAP [24], or estimating neighborhoods in LLE via selection of data objects that are unlikely to be outliers (noise) [13]. Similarly, graph embedding has also been explored with respect to issues such as the scale of analysis and determining accurate groups in the data [25]. However, all of these methods require an exhaustive search of the parameter space in order to best solve the specific problem being addressed. Alternatively, one may utilize class information within the supervised variants [26,27] of ISOMAP and LLE which attempt to construct weighted neighborhood graphs that explicitly preserve class information while embedding the data.

#### Learning in the context of dimensionality reduction

The application of classification theory to DR has begun to be explored recently. Athitsos et al presented a nearest neighbor retrieval method known as BoostMap [28], in which distances from different reference objects are combined via boosting. The problem of selecting and weighting the most relevant distances to reference objects was posed in terms of classification in order to utilize the Adaboost algorithm [16], and BoostMap was shown to improve the accuracy and speed of overall nearest neighbor discovery compared to traditional methods. DR has also previously been formulated in terms of maximizing the entropy [29] or via a simultaneous dimensionality reduction and regression methodology involving Bayesian mixture modeling [30]. The goal in such methods is to probabilistically estimate the relationships between points based on objective functions that are dependent on the data labels [29]. These methods have been demonstrated in the context of application of PCA to non-linear datasets [30]. More recently, multi-view learning algorithms [31] have attempted to address the problem of improving the learning ability of a system by considering several disjoint subsets of features (views) of the data. The work most closely related to our own is that of [32] in the context of web data mining via multi-view learning. Given that a hidden pattern exists in a dataset, different views of this data are each embedded and transformed such that known domain information (encoded via pairwise link constraints) is preserved within a common frame of reference. The authors then solve for a consensus pattern which is considered the best approximation of the underlying hidden pattern being solved for. A similar idea was examined in [33,34] where 1D projections of image data were co-registered in order to better perform operations such as image-based breathing gating as well as multi-modal registration. Unlike consensus embedding, these algorithms involve explicit transformations of embedding data to a target frame of reference, as well as being semi-supervised in encoding specific link constraints in the data.

#### Intuition and significance of consensus embedding

In this paper we present a novel DR scheme (consensus embedding) that involves first generating and then combining multiple uncorrelated, independent (or *base*) *n*-D embeddings. These base embeddings may be obtained via either linear or non-linear DR techniques being applied to a large *N*-D feature space. Note that we use the terms "uncorrelated, independent" with reference to the method of constructing base embeddings; similar to their usage in ensemble classification literature [18]. Indeed, techniques to generate multiple base embeddings may be seen to be analogous to those for constructing classifier ensembles. In the latter, base classifiers with significant variance can be generated by varying the parameter associated with the classification method (*k *in *k*NN classifiers [35]) or by varying the training data (combining decision trees via Bagging [17]). Previously, a consensus method for LLE was examined in [36] with the underlying hypothesis that varying the neighborhood parameter (*κ*) will effectively generate multiple uncorrelated, independent embeddings for the purposes of constructing a consensus embedding. The combination of such base embeddings for magnetic resonance spectroscopy data was found to result in a low-dimensional data representation which enabled improved discrimination of cancerous and benign spectra compared to using any single application of LLE. In this work we shall consider an approach inspired by random forests [37] (which in turn is a modification of the Bagging algorithm [17]), where variations within the feature data are used to generate multiple embeddings which are then combined via our consensus embedding scheme. Additionally, unlike most current DR approaches which require tuning of associated parameters for optimal performance in different datasets, consensus embedding offers a methodology that is not significantly sensitive to parameter choice or dataset type.

The major contributions of our work are:

■ A novel DR approach which generates and combines embeddings.

■ A largely parameter invariant scheme for dimensionality reduction.

■ A DR scheme easily applicable to a wide variety of pattern recognition problems including image partitioning, data mining, and high dimensional data classification.

The organization of the rest of this paper is as follows. In Section 2 we will examine the theoretical grounding and properties of consensus embedding, followed by algorithms to efficiently implement the consensus embedding scheme. In Section 3 we show the application of consensus embedding in the context of (1) partitioning of synthetic as well as clinical images, and (2) classification of gene-expression studies. Quantitative and qualitative results of this evaluation, as well as discussion of the results and concluding remarks, are presented in Section 4.

## Methods

### Theory of Consensus Embedding

The spirit of consensus embedding lies in the generation and combination of multiple embeddings in order to construct a more stable, stronger result. Thus we will first define various terms associated with embedding construction. Based on these, we can mathematically formalize the concept of generating and combining multiple base embeddings, which will in turn allow us to derive necessary and sufficient conditions that must be satisfied when constructing a consensus embedding. Based on these conditions we will describe the specific algorithmic steps in more detail. Notation that is used in this section is summarized in Table 1.

**Table 1:** Notation and symbols

ℝ*^N^*	High(*N*)-dimensional space	ℝ*^n^*	Low(*n*)-dimensional space
*c*, *d*, *e*	Objects in set *C*	*Z*	Number of unique triplets in *C*

**F**(*c*)	High-dimensional feature vector	**X**(*c*)	Embedding vector

Λ*^cd^*	Pairwise relationship in ℝ*^N^*	*δ^cd^*	Pairwise relationship in ℝ*^n^*

Δ(*c*, *d*, *e*)	Triangle relationship (Defn. 1)	*ψ^ES^*(ℝ*^n^*)	Embedding strength (Defn. 2)

${\hat{\mathbb{R}}}^{n}$	True embedding (Defn. 3)	*^${\hat{\delta }}^{cd}$^*	Pairwise relationship in ${\hat{\mathbb{R}}}^{n}$

${\ddot{\mathbb{R}}}^{n}$	Strong embedding (Defn. 4)	${\dot{\mathbb{R}}}^{n}$	Weak embedding

${\tilde{\mathbb{R}}}^{n}$	Consensus embedding (Defn. 5)	*^${\tilde{\delta }}^{cd}$^*	Pairwise relationship in ${\tilde{\mathbb{R}}}^{n}$

*M*	Number of generated embeddings	*K*	Number of selected embeddings

*R*	Number of objects in *C*	$\tilde{X}\left(c\right)$	Consensus embedding vector

Summary of notation and symbols used in this paper.

#### Preliminaries

An object shall be referred to by its label *c *and is defined as a point in an *N*-dimensional space ℝ*^N^*. It is represented by an *N*-tuple **F**(*c*) comprising its unique *N*-dimensional co-ordinates. In a sub-space ℝ*^n ^*⊂ ℝ*^N ^*such that *n << N*, this object *c *in a set *C *is represented by an *n*-tuple of its unique *n*-dimensional coordinates **X**(*c*). ℝ*^n ^*is also known as the *embedding *of objects *c *∈ *C *and is always calculated via some projection of ℝ*^N^*. For example in the case of ℝ^3^, we can define **F**(*c*) = {*f*_1_, *f*_2_, *f*_3_} based on the co-ordinate locations (*f*_1_, *f*_2_, *f*_3_) on each of the 3 axes for object *c *∈ *C*. The corresponding embedding vector of *c *∈ *C *in ℝ^2 ^will be **X**(*c*) = {*e*_1_, *e*_2_} with co-ordinate axes locations (*e*_1_, *e*_2_). Note that in general, determining the target dimensionality (*n*) for any ℝ*^N ^*may be done by a number of algorithms such as the one used in this work [38].

The notation Λ*^cd^*, henceforth referred to as the *pairwise relationship*, will represent the relationship between two objects *c*, *d *∈ *C *with corresponding vectors **F**(*c*), **F**(*d*) ∈ ℝ*^N^*. Similarly, the notation *δ^cd ^*will be used to represent the pairwise relationship between two objects *c*, *d *∈ *C *with embedding vectors **X**(*c*), **X**(*d*) ∈ ℝ*^n^*. We assume that this relationship satisfies the three properties of a metric (e.g. Euclidean distance). Finally, a triplet of objects *c*, *d*, *e *∈ *C *is referred to as an *unique triplet *if *c *≠ *d*, *d *≠ *e*, and *c *≠ *e*. Unique triplets will be denoted simply as (*c*, *d*, *e*).

#### Definitions

**Definition 1 ***The function *Δ *defined on a unique triplet *(*c*, *d*, *e*) *is called a triangle relationship*, Δ(*c*, *d*, *e*)*, if when *Λ*^cd ^<*Λ*^ce ^and *Λ*^cd ^<*Λ*^de^, then δ^cd ^< δ^ce ^and δ^cd ^< δ^de^*.

For objects *c*, *d*, *e *∈ *C *whose relative pairwise relationships in ℝ*^N ^*are preserved in ℝ*^n^*, the triangle relationship Δ(*c*, *d*, *e*) = 1. For ease of notation, the triangle relationship Δ(*c*, *d*, *e*) will be referred to as Δ where appropriate. Note that for a set of *R *unique objects (*R *= |*C*|, |.| is cardinality of a set), $Z=\frac{R!}{3!\left(R-3\right)!}$ unique triplets may be formed.

**Definition 2 ***Given Z unique triplets *(*c*, *d*, *e*) ∈ *C and an embedding *ℝ*^n ^of all objects c*, *d*, *e *∈ *C, the associated embedding strength *$\psi^{ES}\left({\mathbb{R}}^{n}\right)=\frac{\Sigma_{C}\Delta \left(c,d,e\right)}{Z}$.

The embedding strength (ES) of an embedding ℝ*^n^*, denoted *ψ^ES^*(ℝ*^n^*), is hence the fraction of unique triplets (*c*, *d*, *e*) ∈ *C *for which Δ(*c*, *d*, *e*) = 1.

**Definition 3 ***A true embedding*, ${\hat{\mathbb{R}}}^{n}$, *is an embedding for which *$\psi^{ES}\left({\hat{\mathbb{R}}}^{n}\right)=1$.

A true embedding ${\hat{\mathbb{R}}}^{n}$ is one for which the triangle relationship is satisfied for all unique triplets (*c*, *d*, *e*) ∈ *C*, hence perfectly preserving all pairwise relationships from ℝ*^N ^*to ${\hat{\mathbb{R}}}^{n}$. Additionally, for all objects *c*, *d *∈ *C *in ${\hat{\mathbb{R}}}^{n}$, the pairwise relationship is denoted as ${\hat{\delta }}^{cd}$.

Note that according to Definition 3, the most optimal true embedding may be considered to be the original ℝ*^N ^*itself, i.e. ${\hat{\delta }}^{cd}=\Lambda^{cd}$. However, as ℝ*^N ^*may not be optimal for classification (due to the curse of dimensionality), we are attempting to approximate a true embedding as best possible in *n*-D space. Note that multiple true embeddings in *n*-D space may be calculated from a single ℝ*^N^*; any one of these may be chosen to calculate ${\hat{\delta }}^{cd}$.

Practically speaking, any ℝ*^n ^*will be associated with some degree of error compared to the original ℝ*^N^*. This is almost a given since some loss of information and concomitant error can be expected to occur in going from a high- to a low-dimensional space. We can calculate the probability of pairwise relationships being accurately preserved from ℝ*^N ^*to ℝ*^n ^*i.e. the probability that Δ(*c*, *d*, *e*) = 1 for any unique triplet (*c*, *d*, *e*) ∈ *C *in any ℝ*^n ^*as,

(1) $$p\left(\Delta |c,d,e,{\mathbb{R}}^{n}\right)=\frac{\sum_{C}\Delta \left(c,d,e\right)}{Z}.$$

More details on this formulation may be found in the Appendix. Note that the probability in Equation 1 is binomial as the complementary probability to *p*(Δ|*c*, *d*, *e*, ℝ*^n^*) (i.e. the probability that Δ(*c*, *d*, *e*) ≠ 1 for any unique triplet (*c*, *d*, *e*) ∈ *C *in any ℝ*^n^*) is given by 1 - *p*(Δ|*c*, *d*, *e*, ℝ*^n^*) (in the case of binomial probabilities, event outcomes can be broken down into two probabilities which are complementary, i.e. they sum to 1).

**Definition 4 ***A strong embedding*, ${\ddot{\mathbb{R}}}^{n}$, is an embedding for which $\psi^{ES}\left({\ddot{\mathbb{R}}}^{n}\right)>\theta$.

In other words, a strong embedding is defined as one which accurately preserves the triangle relationship for more than some significant fraction (*θ*) of the unique triplets of objects *c*, *d*, *e *∈ *C *that exist. An embedding ℝ*^n ^*which is not a strong embedding is referred to as a *weak embedding*, denoted as ${\dot{\mathbb{R}}}^{n}$.

We can calculate multiple uncorrelated (i.e. independent) embeddings from a single ℝ*^N ^*which may be denoted as ${\mathbb{R}}_{m}^{n},m\in \left\{1,\ldots ,M\right\}$, where *M *is total number of possible uncorrelated embeddings. Note that both strong and weak embeddings will be present among all of the *M *possible embeddings. All objects *c*, *d *∈ *C *can then be characterized by corresponding embedding vectors $X_{m}\left(c\right),X_{m}\left(d\right)\in {\mathbb{R}}_{m}^{n}$ with corresponding pairwise relationship $\delta_{m}^{cd}$. Given multiple $\delta_{m}^{cd}$, we can form a distribution $p\left(X=\delta_{m}^{cd}\right)$, over all *M *embeddings. Our hypothesis is that the maximum likelihood estimate (MLE) of $p\left(X=\delta_{m}^{cd}\right)$, denoted as ${\tilde{\delta }}^{cd}$, will approximate the true pairwise relationship ${\hat{\delta }}^{cd}$ for objects *c*, *d *∈ *C*.

**Definition 5 ***An embedding *ℝ^*n *^is called a consensus embedding, ${\tilde{\mathbb{R}}}^{n}$, if for all objects $c,d\in C,\delta^{cd}={\tilde{\delta }}^{cd}$.

We denote the consensus embedding vectors for all objects *c *∈ *C *by $\tilde{X}\left(c\right)\in {\tilde{\mathbb{R}}}^{n}$. Additionally, from Equation 1, $p\left(\Delta |c,d,e,{\tilde{\mathbb{R}}}^{n}\right)$ represents the probability that Δ(*c*, *d*, *e*) = 1 for any (*c*, *d*, *e*) ∈ *C *in ${\tilde{\mathbb{R}}}^{n}$.

#### Necessary and sufficient conditions for consensus embedding

While ${\tilde{\mathbb{R}}}^{n}$ is expected to approximate ${\hat{\mathbb{R}}}^{n}$ as best possible, it cannot be guaranteed that $\psi^{ES}\left({\tilde{\mathbb{R}}}^{n}\right)=1$ as this is dependent on how well ${\tilde{\delta }}^{cd}$ approximates ${\hat{\delta }}^{cd}$, for all objects *c*, *d *∈ *C*. ${\tilde{\delta }}^{cd}$ may be calculated inaccurately as a result of considering pairwise relationships derived from weak embeddings, ${\dot{\mathbb{R}}}^{n}$, present amongst the *M *embeddings that are generated. As Proposition 1 and Lemma 1 below demonstrate, in order to ensure that $\psi^{ES}\left({\tilde{\mathbb{R}}}^{n}\right)\to 1$, ${\tilde{\mathbb{R}}}^{n}$ must be constructed from a combination of multiple strong embeddings ${\ddot{\mathbb{R}}}^{n}$ alone, so as to avoid including weak embeddings.

**Proposition 1 ***If K *≤ *M independent, strong embeddings *${\mathbb{R}}_{k}^{n},\;k\in \left\{1,\ldots ,K\right\}$, *with a constant *$p\left(\Delta |c,d,e,{\mathbb{R}}_{k}^{n}\right)$*that *Δ(*c*, *d*, *e*) = 1 *for all *(*c*, *d*, *e*) ∈ *C, are used to calculate *${\tilde{\mathbb{R}}}^{n}$, $\psi^{ES}\left({\tilde{\mathbb{R}}}^{n}\right)\to 1$*as K *→ ∞.

**Proof**. If *K *≤ *M *independent, strong embeddings alone are utilized in the construction of ${\tilde{\mathbb{R}}}^{n}$, then the number of weak embeddings is (*M *- *K*). As Equation 1 represents a binomial probability, $p\left(\Delta |c,d,e,{\tilde{\mathbb{R}}}^{n}\right)$ can be approximated via the binomial formulation of Equation 1 as,

(2) $$p\left(\Delta |c,d,e,{\tilde{\mathbb{R}}}^{n}\right)=\overset{M}{\underset{K=1}{\sum }}\left(\begin{array}{c} M\\ K \end{array}\right)\alpha^{K}{\left(1-\alpha \right)}^{M-K},$$

where $\alpha =p\left(\Delta |c,d,e,{\mathbb{R}}_{k}^{n}\right)$ (Equation 1) is considered to be constant. Based on Equation 2, as *K *→ ∞, $p\left(\Delta |c,d,e,{\tilde{\mathbb{R}}}^{n}\right)\to 1$, which in turn implies that $\psi^{ES}\left({\tilde{\mathbb{R}}}^{n}\right)\to 1$; therefore ${\tilde{\mathbb{R}}}^{n}$ approaches ${\hat{\mathbb{R}}}^{n}$.   □

Proposition 1 demonstrates that for a consensus embedding to be strong, it is sufficient that strong embeddings be used to construct it. Note that as *K *→ *M*, $p\left(\Delta |c,d,e,{\tilde{\mathbb{R}}}^{n}\right)>>p\left(\Delta |c,d,e,{\mathbb{R}}_{k}^{n}\right)$. In other words, if $p\left(\Delta |c,d,e,{\mathbb{R}}_{k}^{n}\right)>\theta ,p\left(\Delta |c,d,e,{\tilde{\mathbb{R}}}^{n}\right)>>\theta$. Based on Equation 1 and Definitions 2, 4, this implies that as *K *→ *M*, $\psi^{Es}\left({\tilde{\mathbb{R}}}^{n}\right)>>\theta$. Lemma 1 below demonstrates the necessary nature of this condition i.e. if weak embeddings are considered when constructing ${\tilde{\mathbb{R}}}^{n}$, $\psi^{Es}\left({\tilde{\mathbb{R}}}^{n}\right)<<\theta$ (it will be a weak embedding).

**Lemma 1 ***If K *≤ *M independent, weak embeddings *${\mathbb{R}}_{k}^{n},k\in \left\{1,\ldots ,K\right\}$, *with *$\psi^{ES}\left({\mathbb{R}}_{k}^{n}\right)\leq \theta$, *are used to calculate *${\tilde{\mathbb{R}}}^{n}$, *then *$\psi^{Es}\left({\tilde{\mathbb{R}}}^{n}\right)<<\theta$.

**Proof**. From Equation 1 and Definitions 2, 4, if $\psi^{ES}\left({\mathbb{R}}_{k}^{n}\right)\leq \theta$, then $p\left(\Delta |c,d,e,{\mathbb{R}}_{k}^{n}\right)\leq \theta$. Substituting $p\left(\Delta |c,d,e,{\mathbb{R}}_{k}^{n}\right)$ in Equation 2, will result in $p\left(\Delta |c,d,e,{\tilde{\mathbb{R}}}^{n}\right)<<\theta$. Thus $\psi^{Es}\left({\tilde{\mathbb{R}}}^{n}\right)<<\theta$, and ${\tilde{\mathbb{R}}}^{n}$ will be weak.   □

Proposition 1 and Lemma 1 together demonstrate the necessary and sufficient nature of the conditions required to construct a consensus embedding: that if a total of *M *base embeddings are calculated from a single ℝ*^N^*, some minimum number of strong embeddings (*K *≤ *M*) must be considered to construct a ${\tilde{\mathbb{R}}}^{n}$ that is a strong embedding. Further, a ${\tilde{\mathbb{R}}}^{n}$ so constructed will have an embedding strength $\psi \left({\tilde{\mathbb{R}}}^{n}\right)$ that will increase significantly as we include more strong embeddings in its computation. Appendix B demonstrates an additional property of ${\tilde{\mathbb{R}}}^{n}$ showing that it preserves information from ℝ*^N ^*with less inherent error than any ℝ*^n ^*used in its construction.

### Algorithms and Implementation

Based on Proposition 1, 3 distinct steps are typically required for calculating a consensus embedding. First, we must generate a number of base embeddings (*M*), the steps for which are described in *CreateEmbed*. We then select for strong embeddings from amongst *M *base embeddings generated, described in *SelEmbed*. We will also discuss criteria for selecting strong embeddings. Finally, selected embeddings are combined to result in the final consensus embedding representation as explained in *CalcConsEmbed*. We also discuss some of the computational considerations of our implementation.

#### Creating n-dimensional data embeddings

One of the requirements for consensus embedding is the calculation of multiple uncorrelated, independent embeddings ℝ*^n ^*from a single ℝ*^N^*. This is also true of ensemble classification systems such as Boosting [16] and Bagging [17] which require multiple uncorrelated, independent classifications of the data to be generated prior to combination. As discussed previously, the terms "uncorrelated, independent" are used by us with reference to the method of constructing embeddings, as borrowed from ensemble classification literature [18]. Similar to random forests [37], we make use of a *feature space perturbation *technique to generate uncorrelated (base) embeddings. This is implemented by first creating *M *bootstrapped feature subsets of *V *features each (every subset *η_m_*, *m *∈ {1, . . . , *M*} containing $\left({}_{V}^{N}\right)$ features, no DR involved). Note, that the number of samples in each *V*-dimensional subset is the same as in the original *N*-dimensional space. Each *V*-dimensional *η_m _*is then embedded in *n*-D space via DR (i.e. projecting from ℝ*^V ^*to ℝ*^n^*). *M *is chosen such that each of *N *dimensions appears in at least one *η_m_*.

**Algorithm ***CreateEmbed*

**Input**: **F**(*c*) ∈ ℝ*^N ^*for all objects *c *∈ *C*, *n*

**Output**: $X_{m}\left(c\right)\in {\mathbb{R}}_{m}^{n},m\in \left\{1,\ldots ,M\right\}$

**Data Structures**: Feature subsets *η_m_*, total number of subsets *M*, number of features in each subset *V*, DR method Φ

begin

   0. *for m *= 1 *to M do*

   1.    Select *V < N *features from ℝ*^N^*, forming subset *η_m_*;

   2.    Calculate $X_{m}\left(c\right)\in {\mathbb{R}}_{m}^{n}$, for all *c *∈ *C *using *η_m _*and method Φ;

   3. *endfor*

end

As discussed in the introduction, multiple methods exist to generate base embeddings, such as varying a parameter associated with a method (e.g. neighborhood parameter in LLE, as shown in [36]) as well as the method explored in this paper (feature space perturbation). These methods are analogous to methods in the literature for generating base classifiers in a classifier ensemble [18], such as varying *k *in *k*NN classifiers (changing associated parameter) [39], or varying the training set for decision trees (perturbing the feature space) [37].

#### Selection of strong embeddings

Having generated *M *base embeddings, we first calculate their embedding strengths $\psi^{ES}\left({\mathbb{R}}_{m}^{n}\right)$ for all ${\mathbb{R}}_{m}^{n},m\in \left\{1,\ldots ,M\right\}$. The calculation of *ψ^ES ^*can be done via performance evaluation measures such as those described below, based on the application and prior domain knowledge. Embeddings for which $\psi^{ES}\left({\mathbb{R}}_{m}^{n}\right)>\theta$ are then selected as strong embeddings, where *θ *is a pre-specified threshold.

**Algorithm ***SelEmbed*

**Input**: $X_{m}\left(c\right)\in {\mathbb{R}}_{m}^{n}$ for all objects *c *∈ *C*, *m *∈ {1, . . . , *M*}

**Output**: $X_{k}\left(c\right)\in {\mathbb{R}}_{k}^{n},k\in \left\{1,\ldots ,K\right\}$

**Data Structures**: A list *Q*, embedding strength function *ψ^ES^*, embedding strength threshold *θ*

begin

   0. *for m *= 1 *to M do*

   1.    Calculate $\psi^{ES}\left({\mathbb{R}}_{m}^{n}\right)$;

   2.    *if *$\psi^{ES}\left({\mathbb{R}}_{m}^{n}\right)>\theta$

   3.       Put *m *in *Q*;

   4.    *endif*

   5. *endfor*

   6. For each element *k *of *Q*, store $X_{k}\left(c\right)\in {\mathbb{R}}_{k}^{n}$ for all objects *c *∈ *C*;

end

Note that while *θ *may be considered to be a parameter which needs to be specified to construct the consensus embedding, we have found in our experiments that the results are relatively robust to variations in *θ*. In general, *θ *may be defined based on the manner of evaluating the embedding strength, as discussed in the next section.

#### Evaluation of embedding strength

We present two performance measures in order to evaluate embedding strength: one measure being supervised and relying on label information; the other being unsupervised and driven by the separability of distinct clusters in the reduced dimensional embedding space. In Experiment 4 we compare the two performance measures against each other to determine their relative effectiveness in constructing a strong consensus embedding.

##### Supervised evaluation of embedding strength

We have demonstrated that embedding strength increases as a function of classification accuracy (Theorem 1, Appendix), implying that strong embeddings will have high classification accuracies. Intuitively, this can be explained as strong embeddings showing greater class separation compared to weak embeddings. Given a binary labeled set of samples *C*, we denote the sets of objects corresponding to the two classes as *S*^+ ^and *S*^-^, such that *C *= *S*^+ ^∪ *S*^- ^and *S*^+ ^∩ *S*^- ^= ∅. When using a classification algorithm that does not consider class labels, we can evaluate classification accuracy as follows:

1. Apply classification algorithm to *C *(embedded in ℝ*^n^*) to find *T *clusters (unordered, labeled set of objects), denoted via ${\hat{\Psi }}_{t},t\in \left\{1,\ldots ,T\right\}$.

2. For each ${\hat{\Psi }}_{t}$

(a) Calculate $DTP=\;|{\hat{\Psi }}_{t}\cap S^{+}|$.

(b) Calculate $DTN=\;|\left(C-{\hat{\Psi }}_{t}\right)\cap S^{-}|$.

(c) Calculate classification accuracy for ${\hat{\Psi }}_{t}$, as $\phi^{Acc}\left({\hat{\Psi }}_{t}\right)=\frac{DTP+DTN}{\left|s^{+}\cup s^{-}\right|}$.

3. Calculate classification accuracy of ℝ*^n ^*as $\phi^{Acc}\left({\mathbb{R}}^{n}\right)=\underset{T}{\max}\left[\phi^{Acc}\left({\hat{\Psi }}_{t}\right)\right]$.

As classification has been done without considering label information, we must evaluate which of the clusters so obtained shows the greatest overlap with *S*^+ ^(the class of interest). We therefore consider the classification accuracy of the cluster showing the most overlap with *S*^+ ^as an approximation of the embedding strength of ℝ*^n^*, i.e. *ψ^ES^*(ℝ*^n^*) ≈ *ϕ^Acc^*(ℝ*^n^*).

##### Unsupervised evaluation of embedding strength

We utilize a measure known as the R-squared index (RSI), based off cluster validity measures [40], which can be calculated as follows:

1. Apply classification algorithm to *C *(embedded in ℝ*^n^*) to find *T *clusters (unordered, labeled set of objects), denoted via ${\hat{\Psi }}_{t},t\in \left\{1,\ldots ,T\right\}$.

2. Calculate $SST=\sum_{j=1}^{n}\left[\sum_{i=1}^{R}{\left(X\left(c_{i}\right)-X\left(c_{j}\right)\right)}^{2}\right]$ (where **X**(*c_j_*) is the mean of data values in the *j^th ^*dimension).

3. Calculate $SSB=\sum_{\begin{array}{c} j=1\cdot \cdot \cdot n\\ t=1\cdot \cdot \cdot T \end{array}}\left[\sum_{i=1}^{\left|{\hat{\Psi }}_{t}\right|}{\left(X\left(c_{i}\right)-X\left(c_{j}\right)\right)}^{2}\right]$.

4. Calculate R-squared index of ℝ*^n ^*as $\phi^{RS}\left({\mathbb{R}}^{n}\right)=\frac{SST-SSB}{SST}$.

RSI may be considered both a measure of the degree of difference between clusters found in a dataset as well as measurement of the degree of homogeneity between them. The value of *ϕ^RS ^*ranges between 0 and 1, where if *ϕ^RS ^*= 0, no difference exists among clusters. Conversely, a value close to *ϕ^RS ^*= 1 suggests well-defined, separable clusters in the embedding space. Note that when using RSI to evaluate embedding strength, it will be difficult to ensure that all selected embeddings are strong without utilizing *a priori *information. In such a case we can attempt to ensure that a significant majority of the embeddings selected are strong, which will also ensure that the consensus embedding ${\tilde{\mathbb{R}}}^{n}$ is strong (based off Proposition 1).

#### Constructing the consensus embedding

Given *K *selected embeddings ${\mathbb{R}}_{k}^{n},k\in \left\{1,\ldots ,K\right\}$, we quantify pairwise relationships between all the objects in each ${\mathbb{R}}_{k}^{n}$ via Euclidean pairwise distances. Euclidean distances were chosen for our implementation as they are well understood, satisfy the metric assumption of the pairwise relationship, as well as being directly usable within the other methods used in this work. Ω*_k _*denotes the ML estimator used for calculating ${\tilde{\delta }}^{cd}$ from *K *observations $\delta_{k}^{cd}$ for all objects *c*, *d *∈ *C*.

Algorithm *CalcConsEmbed*

**Input**: $X_{k}\left(c\right)\in {\mathbb{R}}_{k}^{n}$ for all objects *c *∈ *C*, *k *∈ {1, . . . , *K*}

**Output**: $\tilde{X}\left(c\right)\in {\tilde{\mathbb{R}}}^{n}$

**Data Structures**: Confusion matrix *W*, ML estimator Ω, projection method *γ*

begin

   0. *for k *= 1 *to K do*

   1.    Calculate *W_k_*(*i*, *j*) = ||**X***_k_*(*c*) - **X***_k_*(*d*)||_2 _for all objects *c*, *d *∈ *C *with indices *i*, *j*;

   2. *endfor*

   3. Apply normalization to all *W_k_*, *k *∈ {1, . . . , *K*};

   4. Obtain $\tilde{W}\left(i,j\right)=\Omega_{k}\left[W_{k}\left(i,j\right)\right]\forall c,d\in C$;

   5. Apply projection method *γ *to $\tilde{W}$ to obtain final consensus embedding ${\tilde{\mathbb{R}}}^{n}$;

end

Corresponding entries across all *W_k _*(after any necessary normalization) are used to estimate ${\tilde{\delta }}^{cd}$ (and stored in $\tilde{W}$). In our implementation, we have used the median as the ML estimator as (1) the median is less corruptible to outliers, and (2) the median and the expectation are interchangeable if one assumes a normal distribution [41]. In Section 3 we compare classification results using both the mean and median individually as the ML estimator. We apply a projection method *γ*, such as multi-dimensional scaling (MDS) [21], to the resulting $\tilde{W}$ to embed the objects in ${\tilde{\mathbb{R}}}^{n}$ while preserving the pairwise distances between all objects *c *∈ *C*. The underlying intuition for this final step is based on a similar approach adopted in [15] where MDS was applied to the co-association matrix (obtained by accumulating multiple weak clusterings of the data) in order to visualize the clustering results. As $\tilde{W}$ is analogous to the co-association matrix, the projection method *γ *will allow us to construct the consensus embedding space ${\tilde{\mathbb{R}}}^{n}$.

One can hypothesize that $\tilde{W}$ is an approximation of distances calculated in the original feature space. Distances in the original feature space can be denoted as $Ŵ\left(i,j\right)=\;||F\left(c\right)-F\left(d\right)|{|}_{2}\forall c,d\in C$ with indices *i*, *j*. An alternative approach could therefore be to calculate  in the original feature space and apply *γ *to it instead. However, noise artifacts in the original feature space may prevent it from being truly optimal for analysis [11]. As we will demonstrate in Section 3, simple DR, as well as consensus DR, provide superior representations of the data (by accounting for noise artifacts) as compared to using the original feature space directly.

#### Computational efficiency of Consensus Embedding

The most computationally expensive operations in consensus embedding are (1) calculation of multiple uncorrelated embeddings (solved as an eigenvalue problem in *O*(*n*^3^) time for *n *objects), and (2) computation of pairwise distances between all the objects in each strong embedding space (computed in time *O*(*n*^2^) for *n *objects). A slight reduction in both time and memory complexity can be achieved based on the fact that distance matrices will be symmetric (hence only the upper triangular need be calculated). Additionally, multiple embeddings and distance matrices can be computed via code parallelization. However these operations still scale polynomially based on the number of objects *n*.

To further reduce the computational burden we embed the consensus embedding paradigm within an intelligent sub-sampling framework. We make use of a fast implementation [42] of the popular mean shift algorithm [43] (MS) to iteratively represent data objects via their most representative cluster centers. As a result, the space retains its original dimensionality, but now comprises only some fractional number (*n*/*t*) of the original objects. These *n*/*t *objects are used in the calculations of consensus embedding as well as for any additional analysis. A mapping (*Map*) is retained from all *n *original objects to the final *n*/*t *representative objects. We can therefore map back results and analyses from the lowest resolution (*n*/*t *objects) to the highest resolution (*n *objects) easily. The fewer number of objects (*n*/*t << n*) ensures that consensus embedding is computationally feasible. In our implementation, *t *was determined automatically based on the number of stable cluster centers detected by MS.

Algorithm *ConsEmbedMS*

**Input**: **F**(*c*) ∈ ℝ*^N ^*for all objects *c *∈ *C*, *n*

**Output**: $\tilde{X}\left(c\right)\in {\tilde{\mathbb{R}}}^{n}$

**Data Structures**: Reduced set of objects $\bar{c}\in \bar{C}$

begin

   0. Apply MS [42] to ℝ*^N ^*resulting in ${\bar{\mathbb{R}}}^{N}$ for sub-sampled set of objects $\bar{c}\in \bar{C}$;

   1. Save *Map *from sub-sampled set of objects $\bar{c}\in \bar{C}$ to original set of objects *c *∈ *C*;

   2. $X_{m}\left(\bar{c}\right)=CreateEmbed\left(F\left(\bar{c}\right)|\eta_{m},\;\Phi ,\;M,\;V\right),\forall m\in \left\{1,\;\ldots ,\;M\right\}$;

   3. $X_{k}\left(\bar{c}\right)=SelEmbed\left(X_{m}\left(\bar{c}\right)|Q,\;\psi ,\;\theta \right),\forall k\in \left\{1,\;\ldots ,\;K\right\},\forall m\in \left\{1,\;\ldots ,\;M\right\}$;

   4. $\tilde{X}\left(\bar{c}\right)=CalcConsEmbed\left(X_{k}\left(\bar{c}\right)|W,\;\Omega ,\;\gamma \right),\forall k\in \left\{1,\;\ldots ,\;K\right\}$;

   5. Use MS and *Map *to calculate $\tilde{X}\left(c\right)\in {\tilde{\mathbb{R}}}^{n}$ from $\tilde{X}\left(\bar{c}\right)\in {\bar{\mathbb{R}}}^{n}$ for all objects *c *∈ *C*;

end

For an MRI image comprising 5589 pixels (objects) for analysis, the individual algorithms *CreateEmbed*, *SelEmbed *and *CalcConsEmbed *took 121.33, 12.22, and 35.75 seconds respectively to complete (on average). By implementing our mean-shift optimization it took only 119 seconds (on average) for *ConsEmbedMS *to complete analysis of an MRI image comprising between 15,000 and 40,000 pixels (objects); a calculation that would have been computationally intractable otherwise. All experiments were conducted using MATLAB 7.10 (Mathworks, Inc.) on a 72 GB RAM, 2 quad core 2.33 GHz 64-bit Intel Core 2 processor machine.

## Experimental Design for Evaluating Consensus Embedding

### Dataset description

The different datasets used in this work included: (1) synthetic brain image data, (2) clinical prostate image data, and (3) gene-expression data (comprehensively summarized in Table 2). The overarching goal in each experiment described was to determine the degree of improvement in class-based separation via the consensus embedding representation as compared to alternative representations (quantified in terms of classification accuracy). Note that in the case of prostate images as well as gene-expression data we have tested the robustness of the consensus embedding framework via the use of independent training and testing sets.

**Table 2:** Datasets

Datasets	Description	Features
Synthetic brain MRI images	10 slices (109 × 131 comprising 5589 pixels), 6 noise levels (0%, 1%, 3%, 5%, 7%, 9%) 3 RF inhomogeneity levels (0%, 20%, 40%)	Haralick (14)

Prostate MRI images	16 slices, 2 datasets (256 × 256 comprising 15,000-40,000 pixels)	Haralick, 1st order statistical (38)

Gene-Expression data:		
Prostate Tumor	102 training, 34 testing, 12,600 genes	
Breast Cancer Relapse	78 training, 19 testing, 24,481 genes	300 most class-
Lymphoma	38 training, 34 testing, 7130 genes	informative genes
Lung Cancer	32 training, 149 testing, 12,533 genes	

Image and gene-expression datasets used in our experiments.

In the case of image data (brain, prostate MRI), we have derived texture features [44] on a per-pixel basis from each image. These features are based on calculating statistics from a gray level intensity co-occurrence matrix constructed from the image, and were chosen due to previously demonstrated discriminability between cancerous and non-cancerous regions in the prostate [45] and different types of brain matter [46] for MRI data. Following feature extraction, each pixel *c *in the MR image is associated with a *N *dimensional feature vector **F**(*c*) = [*f_u_*(*c*)|*u *∈ {1, . . . , *N*}] ∈ ℝ*^N^*, where *f_u_*(*c*) is the response to a feature operator for pixel *c*. In the case of gene-expression data, every sample *c *is considered to be associated with a high-dimensional gene-expression vector, also denoted **F**(*c*) ∈ ℝ*^N^*.

DR methods utilized to reduce ℝ*^N ^*to ℝ*^n ^*were graph embedding (GE) [6] and PCA [2]. These methods were chosen in order to demonstrate instantiations of consensus embedding using representative linear and non-linear DR schemes. Additionally, these methods have been leveraged both for segmentation as well as classification of similar biomedical image and bioinformatics datasets in previous work [47,48]. The dimensionality of the embedding space, *n*, is calculated as the intrinsic dimensionality of ℝ*^N ^*via the method of [38]. To remain consistent with notation defined previously, the result of DR on **F**(*c*) ∈ ℝ*^N ^*is denoted **X**_Φ_(*c*) ∈ ℝ*^n^*, while the result of consensus DR will be denoted ${\tilde{X}}_{\Phi }\left(c\right)\in {\tilde{\mathbb{R}}}^{n}$. The subscript Φ corresponds to the DR method used, Φ ∈ {*GE*, *PCA*}. For ease of description, the corresponding classification results are denoted Ψ(**F**), Ψ(**X**_Φ_), $\Psi \left({\tilde{X}}_{\Phi }\right)$, respectively.

### Experiment 1: Synthetic MNI brain data

Synthetic brain data [49] was acquired from BrainWeb^1^, consisting of simulated proton density (PD) MRI brain volumes at various noise and bias field inhomogeneity levels. Gaussian noise artifacts have been added to each pixel in the image, while inhomogeneity artifacts were added via pixel-wise multiplication of the image with an intensity non-uniformity field. Corresponding labels for each of the separate regions within the brain, including white matter (WM) and grey matter (GM), were also available. Images comprising WM and GM alone were obtained from 10 sample slices (ignoring other brain tissue classes). The objective was to successfully partition GM and WM regions on these images across all 18 combinations of noise and inhomogeneity, via pixel-level classification (an application similar to Figure 1). Classification is done for all pixels *c *∈ *C *based on each of,

(i) the high-dimensional feature space **F**(*c*) ∈ ℝ*^N^*, *N *= 14,

(ii) simple GE on **F**(*c*), denoted **X***_GE_*(*c*) ∈ ℝ*^n^*, *n *= 3,

(iii) multi-dimensional scaling (MDS) on distances calculated directly in ℝ*^N^*, denoted as **X***_MDS_*(*c*) ∈ ℝ*^n^*, *n *= 3 (alternative to consensus embedding, explained in Section 2),

(iv) consensus embedding, denoted ${\tilde{X}}_{GE}\left(c\right)\in {\tilde{\mathbb{R}}}^{n}$, *n *= 3.

The final slice classification results obtained for each of these spaces are denoted as Ψ(**F**), Ψ(**X***_GE_*), Ψ(**X***_MDS_*), $\Psi \left({\tilde{X}}_{GE}\right)$, respectively.

### Experiment 2: Comparison of ML estimators in consensus embedding

For the synthetic brain data [49], over all 18 combinations of noise and inhomogeneity and over all 10 images, we compare the use of mean and median as ML estimators in *CalcConsEmbed*. This is done by preserving outputs from *SelEmbed *and only changing the ML estimator in the *CalcConsEmbed*. We then compare classification accuracies for detection of white matter in each of the resulting consensus embedding representations, ${\tilde{X}}_{GE}^{Med}$ and ${\tilde{X}}_{GE}^{Mean}$ (superscript denotes choice of ML estimator).

### Experiment 3: Clinical prostate MRI data

Two different prostates were imaged *ex vivo *using a 4 Tesla MRI scanner following surgical resection. The excised glands were then sectioned into 2D histological slices which were digitized using a whole slide scanner. Regions of cancer were determined via Haemotoxylin and Eosin (H&E) staining of the histology sections. The cancer areas were then mapped onto corresponding MRI sections via a deformable registration scheme [50]. Additional details of data acquisition are described in [45].

For this experiment, a total of 16 4 Tesla *ex vivo *T2-weighted MRI and corresponding digitized histology images were considered. The purpose of this experiment was to accurately identify cancerous regions on prostate MRI data via pixel-level classification, based on exploiting textural differences between diseased and normal regions on T2-weighted MRI [45]. For each MRI image, *M *embeddings, ${\mathbb{R}}_{m}^{n},m\in \left\{1,\ldots ,M\right\}$, were first computed (via *CreateEmbed*) along with their corresponding embedding strengths $\psi \left({\mathbb{R}}_{m}^{n}\right)$ (based on clustering classification accuracy). Construction of the consensus embedding was performed via a supervised cross-validation framework, which utilized independent training and testing sets for selection of strong embeddings (*SelEmbed*). The algorithm proceeds as follows,

(a) Training (*S^tr^*) and testing (*S^te^*) sets of the data (MRI images) were created.

(b) For each element (image) of *S^tr^*, strong embeddings were identified based on $\theta =0.15\times \underset{M}{\max}\left[\psi \left({\mathbb{R}}_{m}^{n}\right)\right]$.

(c) Those embeddings voted as being strong across all the elements (images) in *S^tr ^*were then identified and selected.

(d) For the data (images) in *S^te^*, corresponding embeddings were then combined (via *CalcConsEmbed*) to yield the final consensus embedding result.

A leave-one-out cross-validation strategy was employed in this experiment. A comparison is made between the pixel-level classifications for (1) simple GE denoted as Ψ(**X***_GE_*), and (2) consensus GE denoted as $\Psi \left({\tilde{X}}_{GE}\right)$.

### Experiment 4: Gene-expression data

Four publicly available binary class gene-expression datasets were obtained^2 ^with corresponding class labels for each sample [4]; the purpose of the experiment being to differentiate the two classes in each dataset. This data comprises the gene-expression vectorial data profiles of normal and cancerous samples for each disease listed in Table 2, where the total number of samples range from 72 to 181 patients and the number of corresponding features range from 7130 to 24,481 genes or peptides. All 4 data sets comprise independent training (*S^tr^*) and testing (*S^te^*) subsets, and these were utilized within a supervised framework for constructing and evaluating the consensus embedding representation.

Prior to analysis, each dataset was first pruned to the 300 most class-informative features based on *t*-statistics as described in [51]. The supervised cross-validation methodology for constructing the consensus embedding using independent training and testing sets is as follows,

(a) First, *CreateEmbed *is run concurrently on data in *S^tr ^*and *S^te^*, such that the same subsets of features are utilized when generating base embeddings for each of *S^tr ^*and *S^te^*.

(b) *SelEmbed *is then executed on base embeddings generated from *S^tr ^*alone, thus selecting strong embeddings from amongst those generated. Strong embeddings were defined based on $\theta =0.15\times \underset{M}{\max}\left[\psi \left({\mathbb{R}}_{m}^{n}\right)\right]$.

(c) Corresponding (selected) embeddings for data in *S^te ^*are then combined within *CalcConsEmbed *to obtain the final consensus embedding vectors denoted as ${\tilde{X}}_{\Phi }\left(c\right)\in {\tilde{\mathbb{R}}}^{n}$, Φ ∈ {*GE*, *PCA*}, *n *= 4.

For this dataset, both supervised (via clustering classification accuracy, superscript *S*) and unsupervised (via RSI, superscript *US*) measures of embedding strength were evaluated in terms of the classification accuracy of the corresponding consensus embedding representations.

In lieu of comparative DR strategies, a semi-supervised variant of GE [52] (termed SSAGE) was implemented, which utilizes label information when constructing the embedding. Within this scheme, higher weights are given to within-class points and lower weights to points from different classes. When running SSAGE, both *S^tr ^*and *S^te ^*were combined into a single cohort of data, and labels corresponding to *S^tr ^*alone were revealed to the SSAGE algorithm.

An additional comparison was conducted against a supervised random forest-based *k*NN classifier operating in the original feature space to determine whether DR provided any advantages in the context of high-dimensional biomedical data. This was implemented by training a *k*NN classifier on each of the feature subsets for *S^tr ^*(that were utilized in *Create Embed*), but without performing DR on the data. Each such *k*NN classifier was then used to classify corresponding data in *S^te^*. The final classification result for each sample in *S^te ^*is based on ensemble averaging to calculate the probability of a sample belonging to the target class. Classifications compared in this experiment were Ψ(**F**), Ψ(**X***_SSGE_*), $\Psi \left({\tilde{X}}_{GE}^{S}\right)$, $\Psi \left({\tilde{X}}_{PCA}^{S}\right)$, $\Psi \left({\tilde{X}}_{GE}^{US}\right)$, $\Psi \left({\tilde{X}}_{PCA}^{US}\right)$, respectively.

### Classification

For image data (brain, prostate MRI), classification was done via replicated *k*-means clustering [15], while for gene-expression data, classification was done via hierarchical clustering [53]. The choice of clustering algorithm was made based on the type of data being considered in each of the different experiments, as well as previous work in the field. Note that both these clustering techniques do not consider class label information while classifying the data, and have been demonstrated as being deterministic in nature (hence ensuring reproducible results). The motivation in using such techniques for classification was to ensure that no classifier bias or fitting optimization was introduced during evaluation. As our experimental intent was purely to examine improvements in class separation offered by the different data representations, all improvements in corresponding classification accuracies may be directly attributed to improved class discriminability in the corresponding space being evaluated (without being dependent on optimizing the technique used for classification).

### Evaluating and visualizing results

To visualize classification results as region partitions on the images (brain, prostate MRI), all the pixels were plotted back onto the image and assigned colors based on their classification label membership. Similar to the partitioning results shown in Figure 1, pixels of the same color were considered to form specific regions. For example, in Figure 1(h), pixels colored green were considered to form the foreground region, while pixels colored red were considered to form the background.

Classification accuracy of clustering results for images as well as gene-expression data can be quantitatively evaluated as described previously (Section 2). Image region partitioning results as well as corresponding classification accuracies of the different methods (GE, PCA, consensus embedding) were used to determine what improvements are offered by consensus embedding.

## Results and Discussion

### Experiment 1: Synthetic MNI Brain data

**Figure 2:**
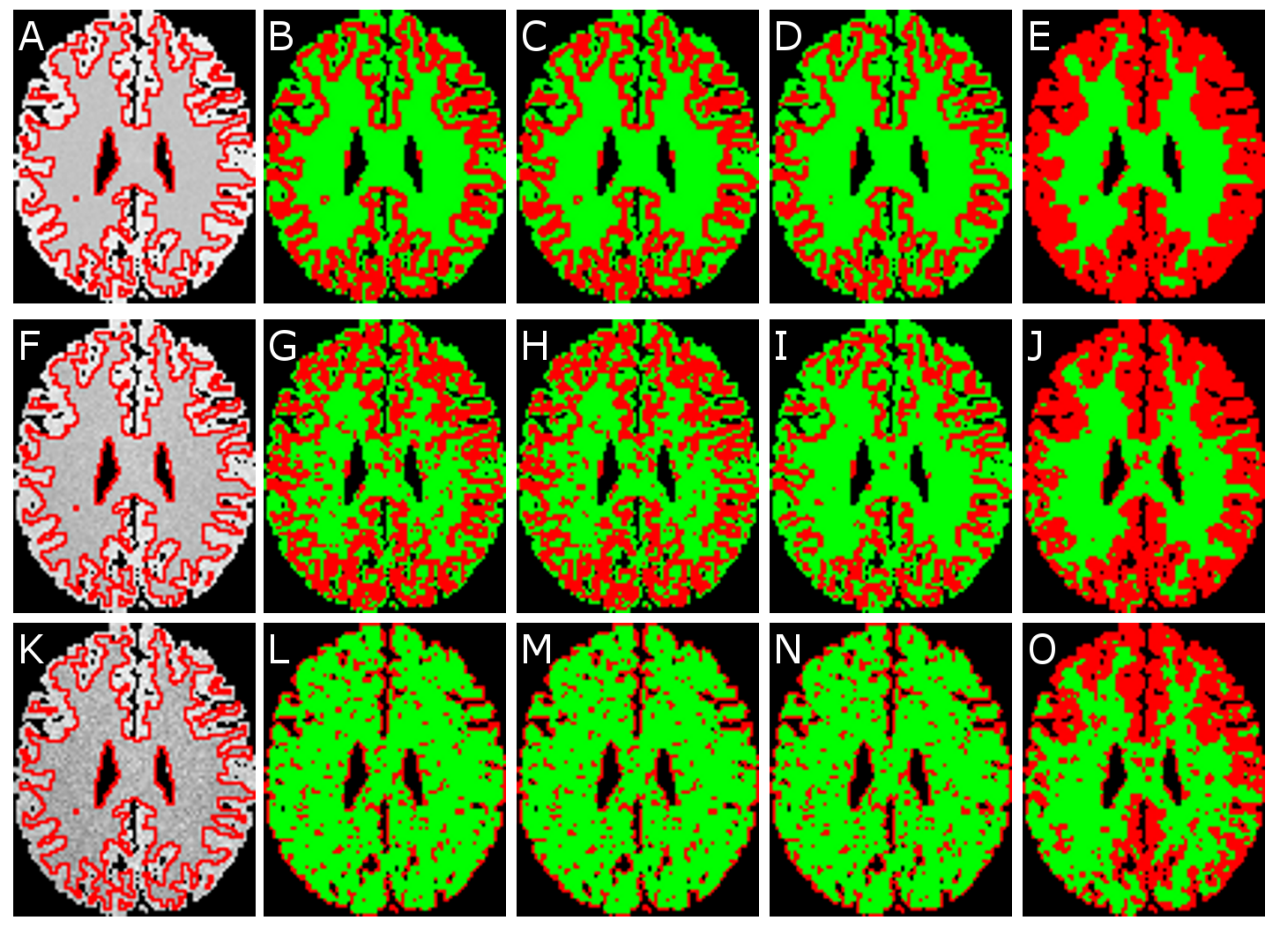
**WM detection results on synthetic BrainWeb data**. Figure 2: Pixel-level WM detection results visualized for one image from the MNI brain MRI dataset, each row corresponding to a different combination of noise and inhomogeneity: (a)-(e) 1% noise, 0% inhomogeneity, (f)-(j) 3% noise, 20% inhomogeneity, (k)-(o) 7% noise, 40% inhomogeneity. The first column shows the original PD MRI image with the ground truth for WM outlined in red, while the second, third, fourth, and fifth columns show the pixel-level WM classification results for Ψ(**F**), Ψ(**X***_MDS_*), Ψ(**X***_GE_*), and $\Psi \left({\tilde{X}}_{GE}\right)$, respectively. The red and green colors in (b)-(e), (g)-(j), (l)-(o) denote the GM and WM regions identified in each result image.

Figure 2 shows qualitative pixel-level WM detection results on MNI brain data for comparisons to be made across 3 different noise and inhomogeneity combinations (out of 18 possible combinations). The original PD MRI image for selected combinations of noise and inhomogeneity with the ground truth for WM superposed as a red contour is shown in Figures 2(a), (f), (k). Note that this is a 2 class problem, and GM (red) and WM (green) region partitions are visualized together in all the result images, as explained previously. Other brain tissue classes were ignored. Comparing the different methods used, when only noise (1%) is added to the data, all three of Ψ(**F**) (Figure 2(b)), Ψ(**X***_MDS_*) (Figure 2(c)), and Ψ(**X***_GE_*) (Figure 2(d)) are only able to identify the outer boundary of the WM region. However, $\Psi \left({\tilde{X}}_{GE}\right)$ (Figure 2(e)) shows more accurate detail of the WM region in the image (compare with the ground truth WM region outlined in red in Figure 2(a)). When RF inhomogeneity (20%) is added to the data for intermediate levels of noise (3%), note the poor WM detection results for Ψ(**F**) (Figure 2(g)), Ψ(**X***_MDS_*) (Figure 2(h)), and Ψ(**X***_GE_*) (Figure 2(i)). $\Psi \left({\tilde{X}}_{GE}\right)$ (Figure 2(j)), however, yields a more accurate WM detection result (compared to the ground truth WM region in Figure 2(f)). Increasing the levels of noise (7%) and inhomogeneity (40%) results in further degradation of WM detection performance for Ψ(**F**) (Figure 2(l)), Ψ(**X***_MDS_*) (Figure 2(m)), and Ψ(**X***_GE_*) (Figure 2(n)). Note from Figure 2(o) that $\Psi \left({\tilde{X}}_{GE}\right)$ appears to fare far better than Ψ(**F**), Ψ(**X***_MDS_*), and Ψ(**X***_GE_*).

**Figure 3:**
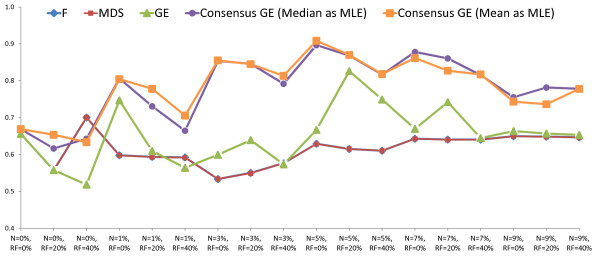
**Trends in WM detection accuracy across Experiments 1 and 2**. Visualization of classification accuracy trends (Tables 3 and 4). $\Psi \left({\tilde{X}}_{GE}\right)$ (consensus embedding) performs significantly better than comparative strategies (original feature space, GE, MDS); using median as ML estimator (purple) may be marginally more consistent than using mean as ML estimator (orange). Ψ(**F**) (blue) and Ψ(**X***_MDS_*) (red) perform similarly (corresponding trends directly superposed on one another).

For each of the 18 combinations of noise and inhomogeneity, we averaged the WM detection accuracies *ϕ^Acc^*(**F**), *ϕ^Acc^*(**X***_MDS_*), *ϕ^Acc^*(**X***_GE_*), ${\phi^{A}}^{cc}\left({\tilde{X}}_{GE}\right)$(calculated as described in Section 2) over all 10 images considered (a total of 180 experiments). These results are summarized in Table 3 (corresponding trend visualization in Figure 3) with accompanying standard deviations in accuracy. Note that ${\phi^{A}}^{cc}\left({\tilde{X}}_{GE}\right)$ shows a consistently better performance than the remaining methods (*ϕ^Acc^*(**F**), *ϕ^Acc^*(**X***_MDS_*), *ϕ^Acc^*(**X***_GE_*)) in 17 out of 18 combinations of noise and inhomogeneity. This trend is also visible in Figure 3.

**Table 3:** WM detection results for synthetic BrainWeb data

Noise	Inhomogeneity	*ϕ^Acc^*(F)	*ϕ^Acc^*(X*_MDS_*)	*ϕ^Acc^*(X*_GE_*)	$\phi^{Acc}\left({\tilde{X}}_{GE}\right)$
	0%	65.55 ± 1.84	65.55 ± 1.84	65.55 ± 1.84	**66.86 **± **2.89**
0%	20%	55.75 ± 1.65	55.75 ± 1.65	55.75 ± 1.65	**61.65 **± **4.58**
	40%	70.03 ± 2.79	**70.08 **± **2.82**	51.84 ± 0.99	64.28 ± 5.93

	0%	59.78 ± 1.31	59.74 ± 1.29	74.71 ± 9.06	**80.62 **± **1.03**
1%	20%	59.36 ± 1.30	59.32 ± 1.33	60.95 ± 8.67	**73.07 **± **8.97**
	40%	59.20 ± 1.12	59.12 ± 1.15	56.38 ± 1.53	**66.46 **± **9.80**

	0%	53.35 ± 1.31	53.39 ± 1.27	59.94 ± 7.00	**85.38 **± **0.75**
3%	20%	55.01 ± 2.92	54.91 ± 3.11	63.88 ± 10.85	**84.61 **± **0.81**
	40%	57.63 ± 1.78	57.71 ± 1.67	57.33 ± 1.38	**79.19 **± **7.56**

	0%	62.90 ± 0.72	62.84 ± 0.66	66.67 ± 10.22	**89.68 **± **1.36**
5%	20%	61.49 ± 1.38	61.49 ± 1.42	82.61 ± 7.39	**86.81 **± **1.38**
	40%	61.02 ± 0.99	61.03 ± 1.09	74.91 ± 9.09	**81.67 **± **1.51**

	0%	64.28 ± 0.71	64.26 ± 0.76	66.95 ± 6.25	**87.81 **± **0.73**
7%	20%	64.07 ± 1.03	64.01 ± 0.96	74.22 ± 10.59	**86.07 **± **1.05**
	40%	64.05 ± 1.19	64.04 ± 1.14	64.44 ± 1.25	**81.53 **± **1.57**

	0%	64.96 ± 0.90	64.94 ± 0.88	66.36 ± 1.66	**75.51 **± **14.35**
9%	20%	64.85 ± 0.97	64.79 ± 0.95	65.68 ± 1.32	**78.18 **± **9.86**
	40%	64.65 ± 0.83	64.63 ± 0.84	65.30 ± 0.74	**77.83 **± **5.00**

Pixel-level WM detection accuracy and standard error averaged over 10 MNI brain images and across 18 combinations of noise and inhomogeneity for each of: (1) Ψ(**F**), (2) Ψ(**X***_MDS_*), (3) Ψ(**X***_GE_*), (4) $\Psi \left({\tilde{X}}_{GE}\right)$ (with median as MLE). Improvements in classification accuracy via $\Psi \left({\tilde{X}}_{GE}\right)$ were found to be statistically significant.

For each combination of noise and inhomogeneity, a paired Students' *t*-test was conducted between ${\phi^{A}}^{cc}\left({\tilde{X}}_{GE}\right)$ and each of *ϕ^Acc^*(**F**), *ϕ^Acc^*(**X***_MDS_*), and *ϕ^Acc^*(**X***_GE_*), with the null hypothesis being that there was no improvement via $\Psi \left({\tilde{X}}_{GE}\right)$ over all 10 brain images considered. $\Psi \left({\tilde{X}}_{GE}\right)$ was found to perform significantly better (*p <*0.05) than all of Ψ(**F**), Ψ(**X***_MDS_*), and Ψ(**X***_GE_*) in 16 out of 18 combinations of noise and inhomogeneity.

Comparing *ϕ^Acc^*(**F**), *ϕ^Acc^*(**X***_MDS_*), and *ϕ^Acc^*(**X***_GE_*), it can be observed that Ψ(**F**) and Ψ(**X***_MDS_*) perform similarly for all combinations of noise and inhomogeneity (note that the corresponding red and blue trend-lines completely overlap in Figure 3). In contrast, Ψ(**X***_GE_*) shows improved performance at every combination of noise and inhomogeneity as compared to either of Ψ(**F**) and Ψ(**X***_MDS_*). $\Psi \left({\tilde{X}}_{GE}\right)$ was seen to significantly improve over all of Ψ(**F**), Ψ(**X***_MDS_*), and Ψ(**X***_GE_*), reflecting the advantages of consensus embedding.

### Experiment 2: Comparison of ML estimators

WM pixel-level detection accuracy results for consensus embedding using two different ML estimators (median and mean) were averaged over all 10 MNI brain images considered and summarized in Table 4, for each of the 18 combinations of noise and inhomogeneity (total of 180 experiments). We see that the accuracy values are generally consistent across all the experiments conducted. No statistically significant difference in classifier performance was observed when using $\Psi \left({\tilde{X}}_{GE}^{Med}\right)$ and $\Psi \left({\tilde{X}}_{GE}^{Mean}\right)$. It would appear that $\Psi \left({\tilde{X}}_{GE}^{Med}\right)$ is less susceptible to higher noise and bias field levels compared to $\Psi \left({\tilde{X}}_{GE}^{Mean}\right)$ (trends in Figure 3).

**Table 4:** Comparing the mean and median as ML estimators within CalcConsEmbed

Noise	Inhomogeneity	$\phi^{Acc}\left({\tilde{X}}_{GE}^{Med}\right)$	$\phi^{Acc}\left({\tilde{X}}_{GE}^{Mean}\right)$
	0%	66.86 ± 2.89	**66.89 **± **2.91**
0%	20%	61.65 ± 4.58	**65.34 **± **4.12**
	40%	**64.28 **± **5.93**	63.39 ± 6.51

	0%	**80.62 **± **1.03**	80.45 ± 1.07
1%	20%	73.07 ± 8.97	**77.81 **± **0.96**
	40%	66.46 ± 9.80	**70.56 **± **7.15**

	0%	85.38 ± 0.75	**85.53 **± **0.84**
3%	20%	**84.61 **± **0.81**	84.49 ± 0.76
	40%	79.19 ± 7.56	**81.37 **± **1.39**

	0%	89.68 ± 1.36	**90.85 **± **1.32**
5%	20%	86.81 ± 1.38	**87.01 **± **1.83**
	40%	81.67 ± 1.51	**81.82 **± **1.32**

	0%	**87.81 **± **0.73**	86.17 ± 6.11
7%	20%	**86.07 **± **1.05**	82.73 ± 8.23
	40%	81.53 ± 1.57	**81.72 **± **1.47**

	0%	**75.51 **± **14.35**	74.32 ± 16.11
9%	20%	**78.18 **± **9.86**	73.63 ± 12.75
	40%	**78.18 **± **9.86**	73.63 ± 12.75

Pixel-level WM detection accuracy and standard error averaged over 10 MNI brain images and for 18 combinations of noise and inhomogeneity (180 experiments) with each of the 2 ML estimators considered in *CalcConsEmbed*: (1) median $\left(\Psi \left({\tilde{X}}_{GE}^{Med}\right)\right)$, (2) $\left(\Psi \left({\tilde{X}}_{GE}^{Mean}\right)\right)$.

### Experiment 3: Clinical Prostate MRI data

**Figure 4:**
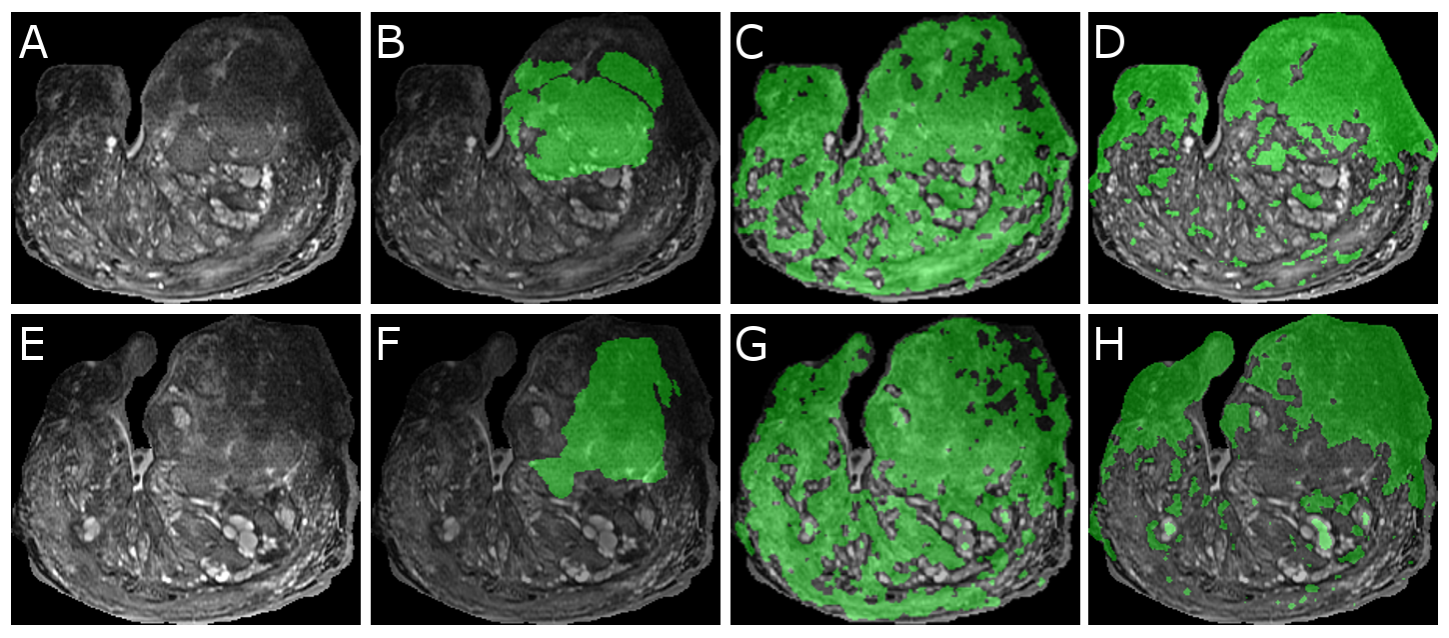
**Prostate cancer detection results on ex vivo MRI data**. (a), (e) 2D sections from 3D prostate MRI data, and (b), (f) corresponding CaP masks superposed on the MRI, obtained via deformable registration with the corresponding histology slice (not shown) [50]. Corresponding CaP detection results via (c), (g) Ψ(**X***_GE_*) (graph embedding), and (d), (h) $\Psi \left({\tilde{X}}_{GE}\right)$ (consensus embedding) are superposed back onto the original MRI sections ((a), (e)). In each of (b)-(d) and (f)-(h), green denotes the CaP segmentation region. Note the significantly fewer false positives in (d) and (h) compared to (c) and (g) respectively.

**Figure 5:**
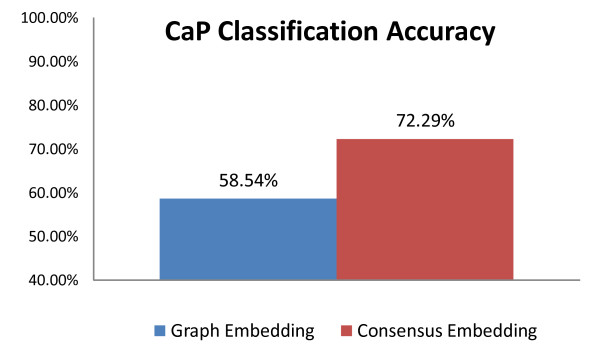
**Prostate cancer detection accuracy on ex vivo MRI data**. Pixel-level classification accuracy in identifying prostate cancer on T2-weighted MRI, averaged over 16 2D MRI slices for Ψ(**X***_GE_*) (blue) and $\Psi \left({\tilde{X}}_{GE}\right)$ (red).

Figure 4 shows qualitative results of the *ConsEmbedMS *algorithm in detecting prostate cancer (CaP) on T2-weighted MRI, each row corresponding to a different 2D MRI image. Comparing the pixel-level CaP detection results (visualized in green) in Figures 4(c) and 4(g) to the green CaP masks in Figures 4(b) and 4(f), obtained by registering the MRI images with corresponding histology images [50] (not shown), reveals that Ψ(**X***_GE_*) results in a large false positive error. In contrast, $\Psi \left({\tilde{X}}_{GE}\right)$ (Figures 4(d) and 4(h)) appears to better identify the CaP region when compared to the ground truth for CaP extent in Figures 4(b) and 4(f). Figure 5 illustrates the relative pixel-level prostate cancer detection accuracies averaged across 16 MRI slices for the 2 methods compared. $\Psi \left({\tilde{X}}_{GE}\right)$ was found to significantly (*p <*0.05) outperform Ψ(**X***_GE_*) in terms of accuracy and specificity of CaP segmentation over all 16 slices considered.

### Experiment 4: Classification of Gene-Expression Data

Table 5 summarizes classification accuracies for each of the strategies compared: supervised consensus-PCA and consensus-GE ($\Psi \left({\tilde{X}}_{PCA}^{S}\right)$, $\Psi \left({\tilde{X}}_{GE}^{S}\right)$, respectively), unsupervised consensus-PCA and consensus-GE ($\Psi \left({\tilde{X}}_{PCA}^{US}\right)$, $\Psi \left({\tilde{X}}_{GE}^{US}\right)$, respectively), SSAGE (Ψ(**X***_SSGE_*)), as well as supervised classification of the original feature space (Ψ(**F**)). These results suggest that consensus embedding yields a superior classification accuracy compared to alternative strategies. We posit that this improved performance is due to the more accurate representation of the data obtained via consensus embedding.

**Table 5:** Classification accuracies for different representation strategies for gene-expression data

Dataset	*ϕ^Acc^*(F)	*ϕ^Acc^*(X*_SSGE_*)	$\phi^{Acc}\left({\tilde{X}}_{PCA}^{S}\right)$	$\phi^{Acc}\left({\tilde{X}}_{PCA}^{US}\right)$	$\phi^{Acc}\left({\tilde{X}}_{GE}^{S}\right)$	$\phi^{Acc}\left({\tilde{X}}_{GE}^{US}\right)$
Prostate Tumor	73.53	73.53	97.06	**100**	**100**	76.47

Breast Cancer Relapse	**68.42**	63.16	63.16	57.89	63.16	57.89

Lung Cancer	89.93	10.07	99.33	96.64	98.66	**100**

Lymphoma	58.82	61.76	**97.06**	76.47	**97.06**	67.65

Classification accuracies for testing cohorts of 4 different binary class gene-expression datasets, comparing (1) supervised random forest classification of original feature space (**F**), (2) unsupervised hierarchical clustering of semi-supervised DR space (**X***_SSGE_*), and (3) unsupervised hierarchical clustering of consensus embedding space $\left({\tilde{X}}_{GE},{\tilde{X}}_{PCA}\right)$.

**Figure 6:**
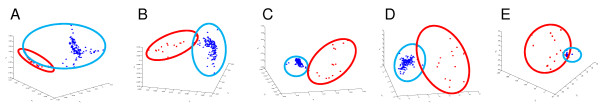
**Visualization of 3D embeddings for gene-expression data (breast cancer)**. 3D visualization of embedding results for lung cancer gene-expression data: (a) **X***_SSGE_*, (b) ${\tilde{X}}_{GE}^{S}$, (c) ${\tilde{X}}_{GE}^{US}$, (d) ${\tilde{X}}_{PCA}^{S}$, (e) ${\tilde{X}}_{PCA}^{US}$. The 3 axes correspond to the primary 3 eigenvalues obtained via different DR methods (SSAGE, consensus-GE and consensus-PCA), while the colors of the objects (red and blue) are based on known class information (cancer and non-cancer, respectively). Note the relatively poor performance of (a) semi-supervised DR compared to (b)-(e) consensus DR. Both supervised ((b) and (d)) and unsupervised ((c) and (e)) consensus DR show relatively consistent separation between the classes with distinct, tight clusters. The best clustering accuracy for this dataset was achieved by (c) unsupervised consensus GE $\left({\tilde{X}}_{GE}^{US}\right)$.

The presence of a large noisy, high-dimensional space was seen to adversely affect supervised classification performance of **F**, which yielded a worse classification accuracy than unsupervised classification (of consensus-GE and consensus-PCA) in 3 out of the 4 datasets. Moreover, semi-supervised DR, which utilized label information to construct **X***_SSGE_*, was also seen to perform worse than consensus embedding (both supervised and unsupervised variants). We posit that this is because SSAGE does not explicitly account for noise, but only modifies the pairwise relationships between points based on label information (possibly exacerbating the effects of noise). By contrast, any label information used by consensus embedding is used to account for noisy samples when approximating the "true" pairwise relationships between points. The difference in the final embedding representations can be visualized in 3D in Figure 6, obtained by plotting all the samples in the lung cancer gene-expression dataset in 3D Eigen space. Note that consensus DR (Figures 6(b)-(e)) shows significantly better separation between the classes with more distinct, tighter clusters as well as fewer false positives compared to SSAGE (Figure 6(a)).

Further, comparing the performance of supervised $\left(\Psi \left({\tilde{X}}_{PCA}^{S}\right),\Psi \left({\tilde{X}}_{GE}^{S}\right)\right)$ and unsupervised $\left(\Psi \left({\tilde{X}}_{PCA}^{US}\right),\Psi \left({\tilde{X}}_{GE}^{US}\right)\right)$ variants of consensus embedding demonstrates comparable performance between them, though a supervised measure of embedding strength shows a trend towards being more consistent. The relatively high performance of $\Psi \left({\tilde{X}}_{PCA}^{US}\right)$ and $\Psi \left({\tilde{X}}_{GE}^{US}\right)$ demonstrate the feasibility of a completely unsupervised framework for consensus embedding.

For both consensus PCA and consensus GE we tested the parameter sensitivity of our scheme by varying the number of feature subsets generated (*M *∈ {200, 500, 1000}) in the *CreateEmbed *algorithm (Tables 6 &7). The relatively low variance in classification accuracy as a function of *M *reflects the invariance to parameters of consensus embedding. No consistent trend was seen in terms of either of consensus-GE or consensus-PCA outperforming the other.

**Table 6:** Variation in classification accuracy as a function of parameters for consensus-PCA on gene-expression data

Dataset	$\phi^{Acc}\left({\tilde{X}}_{PCA}^{S}\right)$			$\phi^{Acc}\left({\tilde{X}}_{PCA}^{US}\right)$		
	
	*M *= 200	*M *= 500	*M *= 1000	*M *= 200	*M *= 500	*M *= 1000
Prostate Tumor	97.06	97.06	97.06	100	100	100

Breast Cancer Relapse	57.89	63.16	57.89	57.89	57.89	52.63

Lung Cancer	99.33	99.33	99.33	96.64	95.97	96.64

Lymphoma	94.12	97.06	97.06	76.47	67.65	61.76

Classification accuracies for testing cohorts of 4 different binary class gene-expression datasets for ${\tilde{X}}_{PCA}^{S}$ and ${\tilde{X}}_{PCA}^{US}$, while varying the number of subsets *M *generated within *CreateEmbed*.

**Table 7:** Variation in classification accuracy as a function of parameters for consensus-GE on gene-expression data

Dataset	$\phi^{Acc}\left({\tilde{X}}_{GE}^{S}\right)$			$\phi^{Acc}\left({\tilde{X}}_{GE}^{US}\right)$		
	
	*M *= 200	*M *= 500	*M *= 1000	*M *= 200	*M *= 500	*M *= 1000
Prostate Tumor	100	100	97.06	76.47	76.47	76.47

Breast Cancer Relapse	57.89	57.89	57.89	57.89	57.89	57.89

Lung Cancer	98.66	98.66	97.99	100	100	90.60

Lymphoma	61.76	97.06	55.88	67.65	67.65	67.65

Classification accuracies for testing cohorts of 4 different binary class gene-expression datasets for ${\tilde{X}}_{GE}^{S}$ and ${\tilde{X}}_{GE}^{US}$, while varying the number of subsets *M *generated within *CreateEmbed*.

## Conclusions

We have presented a novel dimensionality reduction scheme called consensus embedding which can be used in conjunction with a variety of DR methods for a wide range of high-dimensional biomedical data classification and segmentation problems. Consensus embedding exploits the variance within multiple base embeddings and combines them to produce a single stable solution that is superior to any of the individual embeddings, from a classification perspective. Specifically, consensus embedding is able to preserve pairwise object-class relationships from the high- to the low-dimensional space more accurately compared to any single embedding technique. Using an intelligent sub-sampling approach (via mean-shift) and code parallelization, computational feasibility and practicability of our method is ensured.

Results of quantitative and qualitative evaluation in over 200 experiments on toy, synthetic, and clinical images in terms of detection and classification accuracy demonstrated that consensus embedding shows significant improvements compared to traditional DR methods such as PCA. We also compared consensus embedding to using the feature space directly, as well as to using an embedding based on distance preservation directly from the feature space (via MDS [21]), and found significant performance improvements when using consensus embedding. Even though the features and classifier used in these experiments were not optimized for image segmentation purposes, consensus embedding outperforms state-of-the-art segmentation schemes (graph embedding, also known as normalized cuts [6]), differences being statistically significant in all cases. Incorporating spatial constraints via algorithms such as Markov Random Fields [54] could be used to further bolster the image segmentation results via consensus embedding.

In experiments for high-dimensional biomedical data analysis using gene-expression signatures, consensus embedding also demonstrated improved results compared to semi-supervised DR methods (SSAGE [52]). Evaluating these results further illustrates properties of consensus embedding: (1) the consensus of multiple projections improves upon any single projection (via either linear PCA or non-linear GE), (2) the error rate for consensus embedding is not significantly affected by parameters associated with the method, as compared to traditional DR. Finally, the lower performance of a supervised classifier using the original noisy feature space as compared to using the consensus embedding representation demonstrates the utility of DR to obtain improved representations of the data for classification.

It is however worth noting that in certain scenarios, consensus embedding may not yield optimal results. For instance, if very few embeddings are selected for consensus, the improvement in performance via consensus embedding over simple DR techniques may not be as significant. This translates to having a sparsely populated distribution for the estimation of the consensus pairwise relationship, resulting in a lower confidence being associated with it. Such a scenario may arise due to incorrectly specified selection criteria for embeddings; however, it is relatively simple to implement self-tuning for the selection parameter (*θ*). Note we have reported results for a fixed value of *θ *in all our experiments/applications, further demonstrating the robustness of our methodology to choice of parameters.

In this work, consensus embedding has primarily been presented within a supervised framework (using class label information to evaluate embedding strength). Preliminary results in developing an unsupervised evaluation measure using the R-squared cluster validity index [40] are extremely promising. However, additional tuning and testing of the measure is required to ensure robustness.

Another area of future work is developing algorithms for the generating uncorrelated, independent embeddings. This is of great importance as generating truly uncorrelated, independent embeddings will allow us to capture the information from the data better, hence ensuring in an improved consensus embedding result. As mentioned previously, methods to achieve this could include varying the parameter associated with the DR method (e.g. neighborhood parameter in LLE [5]) as well as the feature space perturbation method explored in this paper. These approaches are analogous to methods of generating weak classifiers within a classifier ensemble [18], such as varying the *k *parameter in *k*NN classifiers [39] or varying the training set for decision trees [37]. Note our feature space perturbation method to generate multiple, uncorrelated independent embeddings is closely related to the method used in random forests [37] to generate multiple weak, uncorrelated classifiers. Thus the embeddings we generate, as with the multiple classifiers generated in ensemble classifier schemes, are not intended to be independent in terms of information content, but rather in their method of construction.

The overarching goal of consensus embedding is to optimally preserve pairwise relationships when projecting from high- to low-dimensional space. In this work, pairwise relationships were quantified by us using the popular Euclidean distance metric. This was chosen as it is well understood in the context of these methods used within our algorithm (e.g. the use of MDS). Alternative pairwise relationship measures could include the geodesic distance [7] or the symmetrized Kullback-Leibler divergence [55]. It is important to note that such measures will need to satisfy the properties of a metric to ensure that they correctly quantify both triangle as well as pairwise relationships. We currently use MDS [21] to calculate the final consensus embedding (based on the consensus pairwise distance matrix). We have chosen to use MDS due to ease of computational complexity, but this method could be replaced by a non-linear variant instead. Finally, our intelligent sub-sampling approach to ensure computational feasibility comes with a caveat of the out-of-sample extension problem [56]. We currently handle this using a mapping of results from high to low-resolutions, but are currently identifying more sophisticated solutions. We intend to study these areas in greater detail to further validate the generalizability of consensus embedding.

## Authors' contributions

AM and SV co-conceived the core algorithm and theoretical justifications of consensus embedding. SV further developed, evaluated, and refined the implementation and experiments. AM directed the research and the development of the manuscript. Both authors contributed to writing and editing, and have read and approved the final manuscript.

## Appendix A: Correspondence between Equation 1 and Definition 2

In order to calculate the probability *p*(Δ|*c*, *d*, *e*, ℝ*^n^*), we utilize the traditional formulation of a prior probability,

(3) $$p=\frac{\text{total}\;\text{number}\;\text{of observed instances}}{\text{total}\;\text{number of instances}}.$$

With reference to Equation 1, "instances" are triplets. Therefore Equation 3 becomes,

(4) $$p\left(\Delta \right)=\frac{\text{total number of preserved triplets }\left(\text{i}.\text{e}.\;\Delta =1\right)}{\text{total}\;\text{number possible triplets}}=\frac{\sum_{C}\Delta \left(c,d,e\right)}{Z}.$$

Independent of the above, we intuitively arrived at a mathematical formulation for embedding strength.

The strength of any embedding ℝ*^n ^*will depend on how well pairwise relationships are preserved from ℝ*^N^*.

This in turn can written in terms of the triplet relationship as well,

(5) $$\psi^{EM}\left({\mathbb{R}}^{n}\right)=\frac{\text{total}\;\text{number of preserved triplets}}{\text{total}\;\text{number possible triplets}}=\frac{\sum_{C}\Delta \left(c,d,e\right)}{Z}.$$

## Appendix B: Properties of consensus embedding

The following proposition will demonstrate that ${\tilde{\mathbb{R}}}^{n}$ will have a lower inherent error in its pairwise relationships compared to the strong embeddings ${\mathbb{R}}_{k}^{n},k\in \left\{1,\ldots ,K\right\}$, used in its construction. Note that relevant notation and definitions have been carried over from Section 2.

We first define the mean squared error (MSE) in the pairwise relationship between every pair of objects *c*, *d *∈ *C *in any embedding ℝ*^n ^*with respect to the true pairwise relationships in ${\hat{\mathbb{R}}}^{n}$ as,

(6) $$\varepsilon_{X}={\text{E}}_{cd}{\left({\hat{\delta }}^{cd}-\delta^{cd}\right)}^{2}.$$

where E*_cd _*is the expectation of the squared error in the pairwise relationships in ℝ*^n ^*calculated over all pairs of objects *c*, *d *∈ *C*. We can hence calculate the expected MSE over all *K *base embeddings specified above as,

(7) $$\begin{array}{ll} \varepsilon_{K,X} & ={\text{E}}_{cd}{\left({\hat{\delta }}^{cd}\right)}^{2}-2{\text{E}}_{cd}({\hat{\delta }}^{cd}){\text{E}}_{K}(\delta_{k}^{cd})+{\text{E}}_{cd}{\text{E}}_{K}{\left(\delta_{k}^{cd}\right)}^{2}\\ & \text{Now},{\text{E}}_{K}{\left(\delta_{k}^{cd}\right)}^{2}\geq {\left({\text{E}}_{K}\delta_{k}^{cd}\right)}^{2},\\ & \geq {\text{E}}_{cd}{\left({\hat{\delta }}^{cd}\right)}^{2}-2{\text{E}}_{cd}({\hat{\delta }}^{cd})({\tilde{\delta }}^{cd})+{\text{E}}_{cd}{\left({\tilde{\delta }}^{cd}\right)}^{2}\\ & \geq \varepsilon_{\bar{X}} \end{array}$$

Given *K *observations $\delta_{k}^{cd},k\in \left\{1,\ldots ,K\right\}$ (derived from selected base embeddings ${\mathbb{R}}_{k}^{n}$), we define the pairwise relationship in the consensus embedding ${\tilde{\mathbb{R}}}^{n}$ as ${\tilde{\delta }}^{cd}={\text{E}}_{K}\left(\delta_{k}^{cd}\right)$, where E*_K _*is the expectation of $\delta_{k}^{cd}$ over *K *observations. The MSE in ${\tilde{\delta }}^{cd}$ with respect to the true pairwise relationships in ${\hat{\mathbb{R}}}^{n}$ may be defined as (similar to Equation 6),

(8) $$\varepsilon_{\tilde{X}}={\text{E}}_{cd}{\left({\hat{\delta }}^{cd}-{\tilde{\delta }}^{cd}\right)}^{2},$$

where E*_cd _*is the expectation of the squared error in the pairwise relationships in ${\tilde{\mathbb{R}}}^{n}$ calculated over over all pairs of objects *c*, *d *∈ *C*. It is clear that if for all *c*, *d *∈ *C *that ${\tilde{\delta }}^{cd}={\hat{\delta }}^{cd}$, then ${\tilde{\mathbb{R}}}^{n}$ is also a true embedding.

**Proposition 2 ***Given K independent, strong embeddings*, ${\mathbb{R}}_{k}^{n},k\in \left\{1,\ldots ,K\right\}$, *which are used to construct *${\tilde{\mathbb{R}}}^{n}$, $\varepsilon_{K,X}\geq \varepsilon_{\tilde{X}}$.

**Proof**.

$$\begin{array}{c} \;\;\text{Expanding Equation }7,\\ \varepsilon_{K,X}={\text{E}}_{cd}{\left({\hat{\delta }}^{cd}\right)}^{2}-2{\text{E}}_{cd}\left({\hat{\delta }}^{cd}\right){\text{E}}_{K}\left(\delta_{k}^{cd}\right)+{\text{E}}_{cd}{\text{E}}_{K}{\left(\delta_{k}^{cd}\right)}^{2}\\ \;\;\text{Now},{\text{E}}_{K}{\left(\delta_{k}^{cd}\right)}^{2}\geq {\left({\text{E}}_{K}\delta_{k}^{cd}\right)}^{2},\\ \;\;\geq {\text{E}}_{cd}{\left({\hat{\delta }}^{cd}\right)}^{2}-2{\text{E}}_{cd}\left({\hat{\delta }}^{cd}\right)\left({\tilde{\delta }}^{cd}\right)\;+\;{\text{E}}_{cd}{\left({\tilde{\delta }}^{cd}\right)}^{2}\\ \;\;\geq \varepsilon_{\tilde{X}}\\ \;□ \end{array}$$

Proposition 2 implies that ${\tilde{\mathbb{R}}}^{n}$ will never have a higher error than the maximum error associated with any individual strong embedding ${\mathbb{R}}_{k}^{n},k\in \left\{1,\ldots ,K\right\}$, involved in its construction. However if *ε*_*K*,*X *_is low, $\varepsilon_{\tilde{X}}$ may not significantly improve on it. Similar to Bagging [17] where correlated errors across weak classifiers are preserved in the ensemble result, if the pairwise relationship $\delta_{k}^{cd}$ is incorrect across all *K *embeddings, ${\tilde{\delta }}^{cd}$ will be incorrect as well. However Proposition 2 guarantees that $\varepsilon_{\tilde{X}}$ will never be worse than *ε*_*K*,*X*_.

## Appendix C: Practical implementation of embedding strength

While embedding strength may be seen as a generalized concept for evaluating embeddings, in this work we have examined applications of DR and consensus embedding to classifying biomedical data (Section 3). We now derive a direct relationship between embedding strength and classification accuracy, presented in Theorem 1 below.

For the purposes of the following discussion, all objects *c*, *d*, *e *∈ *C *are considered to be associated with class labels *l*(*c*), *l*(*d*), *l*(*e*) ∈ {*ω*_1_, *ω*_2_}, respectively, such that if *l*(*c*) = *l*(*d*) = *ω*_1 _and *l*(*e*) = *ω*_2 _then Λ*^cd ^<*Λ*^ce ^*and Λ*^cd ^<*Λ*^de^*. Note that *ω*_1_, *ω*_2 _are binary class labels that can be assigned to all objects *c *∈ *C*.

**Definition 6 ***An unique triplet *(*c*, *d*, *e*) ∈ *C with l*(*c*), *l*(*d*), *l*(*e*) ∈ {*ω*_1_, *ω*_2_} *is called a class triplet*, (*c*, *d*, *e*)*_l_, if either l*(*c*) ≠ *l*(*d*)*, or l*(*d*) ≠ *l*(*e*)*, or l*(*c*) ≠ *l*(*e*).

Thus, in a *class triplet *of objects, two out of three objects have the same class label but the third has a different class label, e.g. if *l*(*c*) = *l*(*d*) = *ω*_1 _and *l*(*e*) = *ω*_2_. Further, in the specific case that Δ(*c*, *d*, *e*) = 1 for a class triplet (*c*, *d*, *e*)*_l_*, it will be denoted as Δ*^l^*(*c*, *d*, *e*). For the above example of a class triplet, we know that Λ*^cd ^<*Λ*^ce ^*and Λ*^cd ^<*Λ*^de ^*(see above). If Δ(*c*, *d*, *e*) = 1, *δ^cd ^< δ^ce ^*and *δ^cd ^< δ^de^*. This implies that even after projection from ℝ*^N ^*to ℝ*^n^*, the class-based pairwise relationships within the data are accurately preserved (a classifier can be constructed which will correctly classify objects *c*, *d*, *e *in ℝ*^n^*).

Consider that if $\frac{R}{S}$ objects have class label *ω*_1_, then $\frac{\left(S-1\right)R}{S}$ objects will have class label *ω*_2_. Based on the total number of unique triplets *Z*, the total number of triplets which are not class triplets is,

(9) $$Y=\frac{\frac{R}{S}!}{3!\left(\frac{R}{S}-3\right)!}+\frac{\frac{\left(S-1\right)R}{S}!}{3!\left(\frac{\left(S-1\right)R}{S}-3\right)!}$$

*Y *will be a constant for a given set of objects *C*, and is based on forming unique triplets (*c*, *d*, *e*) where *l*(*c*) = *l*(*d*) = *l*(*e*) (triplets which are not class triplets). *U *= (*Z *- *Y*) will correspond to the number of class triplets that may be formed for set *C*. If all *U *class triplets have Δ*^l^*(*c*, *d*, *e*) = 1, then it is possible to construct *U *classifiers which correctly classify the corresponding objects in these class triplets.

**Definition 7 ***Given U unique class triplets *(*c*, *d*, *e*)*_l _*∈ *C and an embedding *ℝ*^n ^of all objects c*, *d*, *e *∈ *C, the associated classification accuracy *$\phi^{Acc}\left({\mathbb{R}}^{n}\right)=\frac{\sum_{C}\Delta^{l}\left(c,d,e\right)}{U}$

As illustrated previously, class triplets (*c*, *d*, *e*)*_l _*for which Δ*^l^*(*c*, *d*, *e*) = 1 will correspond to those objects which will be classified correctly in ℝ*^n^*. Therefore, the classification accuracy *ϕ^Acc^*(ℝ*^n^*) may simply be defined as the fraction of class triplets (*c*, *d*, *e*)*_l _*∈ *C *for which Δ*^l^*(*c*, *d*, *e*) = 1.

**Theorem 1 ***For any *ℝ*^n^, the corresponding ψ^ES^*(ℝ*^n^*) *increases monotonically as a function of ϕ^Acc^*(ℝ*^n^*).

**Proof**.

$$\begin{array}{c} \;\;\;\;\;\;\;\;\;\text{By definition},\underset{C}{\sum }\Delta \left(c,d,e\right)\geq \underset{C}{\sum }\Delta^{l}\left(c,d,e\right)\\ \text{Dividing by }Z=U+Y\;\text{on either side},\;\psi^{ES}\left({\mathbb{R}}^{n}\right)\geq \frac{\sum_{C}\Delta^{l}\left(c,d,e\right)}{U+Y}\\ \;\;\;\;\;\;\;\;\;\text{Inverting},\frac{1}{\psi^{ES}\left({\mathbb{R}}^{n}\right)}\leq \frac{1}{\phi^{Acc}\left({\mathbb{R}}^{n}\right)}+\frac{Y}{\sum_{C}\Delta^{l}\left(c,d,e\right)} \end{array}$$

As $\frac{Y}{\sum_{C}\Delta^{l}\left(c,d,e\right)}$ is a constant, *ψ^ES^*(ℝ*^n^*) increases monotonically with *ϕ^Acc^*(ℝ*^n^*).   □

Thus an embedding ℝ*^n ^*with a high embedding strength will have a high classification accuracy. Practically, this implies that *ψ^ES^*(ℝ*^n^*) may be estimated via any measure of object-class discrimination such as classification accuracy or cluster-validity measures. We have exploited this relationship in our algorithmic implementation (Section 2).

## Endnotes

^1^http://www.bic.mni.mcgill.ca/brainweb/

^2^These datasets were downloaded from the Biomedical Kent-Ridge Repositories at http://datam.i2r.a-star.edu.sg/datasets/krbd/
